# Stroma-derived miR-214 coordinates tumor dissemination

**DOI:** 10.1186/s13046-022-02553-5

**Published:** 2023-01-13

**Authors:** Francesca Orso, Federico Virga, Daniela Dettori, Alberto Dalmasso, Mladen Paradzik, Aurora Savino, Margherita A. C. Pomatto, Lorena Quirico, Stefania Cucinelli, Martina Coco, Katia Mareschi, Franca Fagioli, Leonardo Salmena, Giovanni Camussi, Paolo Provero, Valeria Poli, Massimiliano Mazzone, Pier Paolo Pandolfi, Daniela Taverna

**Affiliations:** 1 Molecular Biotechnology Center (MBC) “Guido Tarone”, Via Nizza, 52, 10126 Turin, Italy; 2 Dept. Molecular Biotechnology and Health Sciences, University of Turin, Via Nizza, 52, 10126 Turin, Italy; 3 Dept. of Translational Medicine (DIMET), Università del Piemonte Orientale, Novara, Italy; 4 Lab of Tumor Inflammation and Angiogenesis, Center for Cancer Biology (CCB), VIB, Louvain, Belgium; 5 Present Address: Immunobiology Laboratory, Centro Nacional de Investigaciones Cardiovasculares (CNIC), Madrid, Spain; 6 Department of Medical Sciences, University of Turin, Turin, Italy; 7 Paediatric Onco-Haematology Division, Regina Margherita Children’s Hospital, City of Health and Science of Turin, Turin, Italy; 8 Department of Public Health and Paediatrics, University of Turin, Turin, Italy; 9 Princess Margaret Cancer Centre, University Health Network, Toronto, Canada; 10 Center for Omics Sciences, IRCCS San Raffaele Scientific Institute, Milan, Italy; 11 Department of Neurosciences “Rita Levi Montalcini”, University of Turin, Turin, Italy; 12 William N. Pennington Cancer Institute, Renown Health, Nevada System of Higher Education, Reno, NV 89502 USA

**Keywords:** miR-214, Stroma, Extracellular vesicles (EVs), Crosstalk, Metastasis formation, IL-6

## Abstract

**Background:**

Tumor progression is based on a close interaction between cancer cells and Tumor MicroEnvironment (TME). Here, we focus on the role that Cancer Associated Fibroblasts (CAFs), Mesenchymal Stem Cells (MSCs) and microRNAs (miRs) play in breast cancer and melanoma malignancy.

**Methods:**

We used public databases to investigate miR-214 expression in the stroma compartment of primary human samples and evaluated tumor formation and dissemination following tumor cell injections in miR-214 overexpressing (miR-214^over^) and knock out (miR-214^ko^) mice. In addition, we dissected the impact of Conditioned Medium (CM) or Extracellular Vesicles (EVs) derived from miR-214-rich or depleted stroma cells on cell metastatic traits.

**Results:**

We evidence that the expression of miR-214 in human cancer or metastasis samples mostly correlates with stroma components and, in particular, with CAFs and MSCs. We present data revealing that the injection of tumor cells in miR-214^over^ mice leads to increased extravasation and metastasis formation. In line, treatment of cancer cells with CM or EVs derived from miR-214-enriched stroma cells potentiate cancer cell migration/invasion in vitro*.* Conversely, dissemination from tumors grown in miR-214^ko^ mice is impaired and metastatic traits significantly decreased when CM or EVs from miR-214-depleted stroma cells are used to treat cells in culture. Instead, extravasation and metastasis formation are fully re-established when miR-214^ko^ mice are pretreated with miR-214-rich EVs of stroma origin. Mechanistically, we also show that tumor cells are able to induce miR-214 production in stroma cells, following the activation of IL-6/STAT3 signaling, which is then released via EVs subsequently up-taken by cancer cells. Here, a miR-214-dependent pro-metastatic program becomes activated.

**Conclusions:**

Our findings highlight the relevance of stroma-derived miR-214 and its release in EVs for tumor dissemination, which paves the way for miR-214-based therapeutic interventions targeting not only tumor cells but also the TME.

**Supplementary Information:**

The online version contains supplementary material available at 10.1186/s13046-022-02553-5.

## Background

The formation of tumor metastasis is the most frequent outcome of tumor progression and one of the main causes of cancer-related deaths both in melanoma and breast cancer [1]. It is well-known that metastasis formation does not only rely on tumor cell characteristics and behavior, but it is strongly influenced by the crosstalk between tumor and stroma cells [2] in the primary tumor microenvironment (TME), in the blood circulation system during extravasation and when tumor cells disseminate distant organs [3]. Interactions between tumor and stroma cells have been thoroughly studied [4] however, the entire picture is far from being defined. Importantly, many studies underline the relevance of Cancer Associated Fibroblasts (CAFs) and Mesenchymal Stem Cells (MSCs) in tumor spread [5] and propose them as targets for therapies to efficiently fight tumor progression [4, 6].

Extracellular Vesicles (EVs) are produced by tumor and stroma cells, play a major role in the communication between the different cells and are used as mediators of essential signals which favor tumor progression and metastasis formation through their cargos, such as nucleic acids, proteins or lipids [7]. MicroRNAs (miRs) are small non-coding RNAs which inhibit the expression of their cognate target genes and play relevant roles in tumor development and progression [8]. Growing evidence indicates that high levels of miRs are secreted from cells within EVs and transferred to other cell compartments and are crucial regulators of the immune response, chemoresistance and metastasis formation in different cancers [9, 10].

miR-214 expression has been found to be significantly upregulated in malignant melanomas [11–14] and triple negative breast tumors [15] and acts as a pro-metastatic miR by promoting tumor dissemination via a complex pathway which includes transcription factors and adhesion molecules as well as the anti-metastatic miR-148b [11, 12]. In this study, we highlight the essential role of stroma miR-214 in melanoma and breast tumor progression. In fact, CAFs and MSCs of the TME express elevated levels of miR-214 and are able to transfer it to tumor cells via EVs to favor metastatic traits and tumor spreading. Stroma cells produce miR-214 upon tumor cell signals which involve the activation of the IL-6/STAT3 signaling.

## Methods

### Mice

Cre-inducible miR-214 expression construct was generated by cloning a miR-214 expression cassette downstream of a CAGGS promoter and a LoxP-flanked transcription STOP element. This construct was targeted into the mouse Collagen A1 locus using a Flippase (FLP) recombinase-mediated genomic integration. mouse Embryonic Stem cells (mESCs) carrying a single copy of the miR-214^STOP^ construct were identified by resistance to the antibiotic marker hygromycin and Southern blotting. Selected clones were injected into blastocysts to generate pups. To obtain total body overexpressing miR-214^over^, miR-214^STOP^ mice were bred to a Balancer-Cre transgenic strain [16], kindly provided by E. Hirsch. To generate PyMT miR-214^over^ transgenic mice, miR-214^over^ mice were crossed with Mouse Mammary Tumor Virus Polyoma Middle T antigen MMTV-PyMT transgenic mice [17], kindly provided by F. Cavallo’s laboratory, University of Torino, Italy. miR-214^ko^ mice [18] were kindly provided by Eric Olson’s laboratory, UT Southwestern Medical Center, Dallas, USA. The sources of primers used for genotyping are available upon request. All experiments performed with live animals complied with ethical animal care and were approved by the MBC Animal Care Committee and the Italian Ministry of Health (13/2014-PR to DT; 847/2020-PR to DT).

### Cell culture

B16-F10 murine melanoma cells, EO771 mouse mammary tumor cells, NIH3T3 fibroblasts and human bone marrow stromal HS5 cell lines were obtained from The American Type Culture Collection. B16-F10, NIH3T3 and HS5 were maintained in Dulbecco's Modified Eagle's Medium containing 10 mM Glutamax and 4.5 g/L glucose (DMEM Glutamax™, GIBCO Invitrogen Life Technologies, Carlsbad, CA), supplemented with 10% heat-inactivated FCS (Seromed, GmbH), 1 mM sodium pyruvate, 25 mM HEPES pH 7.4 and 100 μg/mL gentamicin (all from GIBCO Invitrogen Life Technologies, Carlsbad, CA). EO771 cells were cultured in RPMI (Roswell Park Memorial Institute) medium containing 10 mM Glutamax and 4.5 g/L glucose (DMEM Glutamax™, GIBCO Invitrogen Life Technologies, Carlsbad, CA), supplemented with 10% heat-inactivated FCS (Seromed, GmbH), 25 mM HEPES pH 7.4 and 100 μg/mL gentamicin (all from GIBCO Invitrogen Life Technologies, Carlsbad, CA). 4175-TGL breast cancer cells were kindly provided by J. Massaguè and maintained as in [19]. MA-2 melanoma cells were a kind gift from Lei Xu and cultured as indicated in [19, 20]. Mesenchymal Stem Cells (MSCs) were derived, characterized and maintained as described in [21]. Cancer Associated Fibroblasts (CAFs) were isolated form PyMT-miR-214^wt^, miR-214^over^ and miR-214^ko^ PyMT tumors. Briefly, mammary tumors (around 0.5 mm^3^) were excised and minced into 2–4 mm fragments, which were then incubated for 3 h with Collagenase A 1 mg/ml. Digested fragments were filtered (70 μm cell strainer) and fibroblast cell populations were enriched through pre-plating and subsequent differential trypsinization. CAFs were maintained in Dulbecco's Modified Eagle's Medium containing 10 mM Glutamax and 4.5 g/L glucose (DMEM Glutamax™, GIBCO Invitrogen Life Technologies, Carlsbad, CA), supplemented with 10% heat-inactivated FCS (Seromed, GmbH), 1 mM sodium pyruvate, 25 mM HEPES pH 7.4 and 100 μg/mL gentamicin (all from GIBCO Invitrogen Life Technologies, Carlsbad, CA). The purity of the isolated population was assessed by Western Blot analysis of the main CAF markers. Tumor Associated Macrophages (TAMs) were derived and maintained as described in [22]. Murine Embryo Fibroblasts (MEFs) were derived and maintained as described in [23]. MEFs Stat3^ko^ and Stat3^wt^ were derived and maintained as described in [24]. For IL-6 experiments, MEFs Stat3^ko^ and Stat3^wt^ were treated with recombinant IL-6 (500 ng/ml) plus soluble receptors (250 ng/ml) as described in [25] for 6 h before RNA extraction. Co-culture experiments were performed as follows. Cultures containing either stroma (MEFs, hMSCs) or melanoma (GFP^+^ B16-F10, GFP^+^ MA-2) cells or both were prepared. A cell preparation with a 1:1 (stroma:tumor) ratio was used. Cells were allowed to attach for 24 h, then media were replaced with fresh DMEM. After 24 h, cells were detached and sorted based on GFP expression as described below.

### Reagents and antibodies

TaqMan® MicroRNA assays for miRNA detection: Hsa-miR-214 ID 002306, Hsa-miR-148b ID000471, Hsa-miR-223 ID 002295 U6 snRNA ID001973, (all from Applied Biosystems, Foster City, USA). TaqMan® Gene expression assays for S100b: ID Mm00485897_m1. Primary antibodies: anti-ITGA5 pAb RM10 kindly provided by G. Tarone laboratory (Molecular Biotechnology Center, University of Torino), anti-CD166/ALCAM mAb MOG/07 (Novocastra Laboratories), anti-E-cadherin mAb #610,182 (BD Transduction Laboratories, Franklin Lakes, USA), anti-N-cadherin pAb ab18203 and anti-αSMA pAb ab15734 (Abcam, Cambridge, United Kingdom), anti-TFAP2C mAb 6E4/4 mAb, H-77 pAb, anti-GAPDH pAb V-18, anti-ACTIN I-19 pAb, anti-Hsp90 mAb F-8 (all from Santa Cruz Biotechnology). Secondary antibodies: HRP-conjugated goat anti-mouse IgG, goat anti-rabbit IgG (all from Santa Cruz Biotechnology, Santa Cruz, CA), goat anti-rabbit IgG Alexa-Fluor-488 (Molecular Probes, Invitrogen Life Technologies, Carlsbad, USA). All antibodies were used at the producer’s suggested concentration.

### Vectors, generation of stable cell lines

Stable miR-214 down-modulation in NIH3T3 and HS5 cells were obtained following transduction of pLenti-CMV-GFP-Puro-miR-214^sponge^ (miR-214sponge) or pLenti-CMV-GFP-Puro (control) expression vectors [19]. GFP^+^ melanoma cells (B16-F10 or MA-2) were obtained following transduction of pLenti-CMV-GFP-Puro lentiviral expression vectors. Lentiviruses were produced according to Trono’s lab protocol (http://tronolab.epfl.ch). Supernatants were harvested 48 h post-transfection, filtered with 0.45 μm filters, diluted and used to infect 3.5 × 10^5^ cells in 6-well plates, in presence of 8 μg/mL Polybrene (Sigma-Aldrich, St Louis, MO). Infected cells underwent puromycin selection to obtain a pure population.

### Fluorescence-activated cell sorting

GFP^+^ B16-F10 subcutaneous tumors were harvested at the end point of the experiment, dissociated with Collagenase A for 1 h and cells sorted based on GFP expression. For co-culture experiments, melanoma (GFP^+^ B16-F10 or GFP^+^ MA-2) and stromal cells (MEFs, hMSCs) were detached and sorted based on GFP expression using a BD FACS Aria III (Becton Dickinson) cell sorter: GFP^+^ tumor fraction; GFP^−^ stroma fraction. Cell pellets were washed and snap-frozen before RNA isolation.

### RNA isolation and qRT-PCR

Total RNA was isolated using TRIzol® Reagent (Invitrogen Life Technologies, Carlsbad, CA). qRT-PCRs for miR detection or gene expression analysis were performed with the indicated TaqMan® MicroRNA or Gene Expression Assays (Applied Biosystems, Foster City, CA) on 10 ng total RNA according to the manufacturer's instructions. qRT-PCRs were carried out using gene-specific primers, using a 7900HT Fast Real Time PCR System (Applied Biosystems, Foster City, CA). Quantitative normalization was performed on RNU6 or RNU44 small nucleolar RNAs expression or 18S expression. The relative expression levels between samples were calculated based on the comparative delta CT (threshold cycle number) method (2-ΔΔCT) using the sample median as reference point as described in [26]. For experiments with Conditioned Medium (CM) or Extracellular Vescicles (EVs), RNA was extracted from tumor or stroma cells following 24-48 h treatments in serum-free medium. When RNA was extracted from tumors or dissected metastases, samples were disrupted with an Ultra TURRAX Homogenizer (IKA®-Werke GmbH) prior Trizol extraction.

### In situ hybridization

Control and miR-214^over^ mouse embryos were collected at 12.5 days post-coitum (E12.5), fixed in 4% PFA/0.1 M Phosphate Buffer (PB, pH 7.4) for 12–16 h, washed in PBS, dehydratated in methanol, processed for paraffin embedding and sectioned at 6 μm. Hybridization was carried out with Digoxigenin (DIG)-labelled Locked Nucleic Acid (LNA) probes, specific for the detection of the mature murine miR-214 (Exiqon, Vedbaek, Denmark) according with manufacturer’s instruction. The sections were hybridized with the probe for 16 h, washed, incubated with an anti-DIG-Alkaline Phosphates (AP) antibody (Roche, GmbH) and developed with NBT-BCIP (Sigma-Aldrich, St Louis, MO). To control the efficiency of the procedure and RNA preservation, the adjacent sections were hybridized with a specific probe for the U6 small nucleolar RNA.

### Northern blot analysis

Total RNA was isolated from miR-214^wt^ and miR-214^over^ embryos collected at 12.5 days post-coitum (E12.5) using TRIzol® Reagent (Invitrogen Life Technologies, Carlsbad, CA) according to manufacturer’s instruction. 25 μg of total RNA were resolved on 12.5% (w/v) TBE–Urea–polyacrylamide gel electrophoresis, transferred to a Hybond N + membrane (GE Healthcare Life Sciences, Piscataway, NJ, USA) and UV crosslinked to membrane. The filter was hybridized overnight at 45 °C with a specific miR-214 digoxigenin-labeled LNA Detection probe (Exiqon, Vedbaek, Denmark), washed and visualized with a specific DIG antibody (1: 10,000) using the DIG Nucleic Acid Detection kit, according to manufacturer’s instructions (all from Roche, GmbH). The filter was then stripped and re-probed overnight at 45 °C using a specific U6 digoxigenin-labeled LNA Detection probe (Exiqon, Vedbaek, Denmark).

### Protein preparation and immunoblotting

Total protein extracts were obtained using a boiling buffer containing 0.125 M Tris/HCl, pH 6.8 and 2.5% Sodium Dodecyl Sulphate (SDS) (Sigma-Aldrich, St Louis, MO). 20–30 µg of proteins were separated by SDS polyacrylamide gel electrophoresis (PAGE) and electroblotted onto nitrocellulose membranes (BioRad). Membranes were blocked in 5% non-fat milk PBS-Tween 0.1% buffer for 1 h at 37 °C, then incubated with appropriate primary and secondary antibodies in PBS-Tween 0.1% buffer, respectively, overnight at 4 °C or for 1 h at room temperature and developed using Chemidoc Touch Imaging System (Bio Rad). For experiments in the presence of Extracellualr Vescicles (EVs), proteins were extracted from tumor cells previously treated with stroma EVs (5000 EVs/cell) for 24-48 h in serum-free medium.

### Conditioned medium (CM) from stroma and tumor cells

miR-214^wt^ and miR-214^over^ MEFs or CAFs and HS5 or NIH3T3 control and miR-214^sponge^ or B16-F10, MA-2 cells were grown to sub-confluence and treated for 48 h with serum-free medium to obtain the corresponding CM to use on recipient cells which were then kept with the CM for 24-48 h, before RNA/protein extractions or biological experiments. To obtain Extracellular Vescicle-depleted CM (EVs-depleted), CM was harvested and centrifuged for 30 min at 3,000 g to remove cell debris and apoptotic bodies. After that, the supernatant was centrifuged for 2 h at 100,000 g, 4 °C using the Beckman Coulter Optima L‐100 K Ultracentrifuge with the rotor type 45 Ti 45,000 rpm. The supernatant was then collected and centrifuged again for 2 h at the same conditions to remove remaining EVs. For anti-IL-6R and anti-IL-6 blocking antibody experiments, CAFs were treated with EV-depleted B16-F10-derived CM plus 50 μg/ml of anti-mouse IL-6R (rat MAb 15A7 clone) or 10 μg/ml of anti-mouse IL-6 (rat Mab clone MP5-20F3, BioXCell), respectively, or control IgG (Thermo Fisher Scientific) for 6 h, before RNA extraction.

### Extracellular Vesicle (EV) isolation and characterization

Isolation of EVs was performed as described in [27]. Briefly, sub-confluent miR-214^wt^ and miR-214^over^ MEFs or CAFs or miR-214^sponge^ and control NIH3T3 or HS5 cells were cultured in serum‐free DMEM for 18 h. The medium was then centrifuged for 30 min at 3,000 g to remove cell debris and apoptotic bodies. After that, the supernatant was centrifuged for 2 h at 100,000 g, 4 °C using the Beckman Coulter Optima L‐100 K Ultracentrifuge with the rotor type 45 Ti 45,000 rpm. The pellet of EVs obtained was resuspended in DMEM supplemented with 1% DMSO. Suspension of isolated EVs was then stored at − 80 °C until further use. Alternatively, EV pellets were resuspended in Trizol Reagent for RNA extraction. EVs were analyzed using the NanoSight NS300 system (Malvern Instruments, Ltd). For isolation of EVs from mouse, blood was collected, plasma was derived and Exoquick™ reagent (System Bioscience, Palo Alto, CA) was used according to manufacturer’s instructions. Immediately after EV isolation, RNA was extracted using the miRNeasy Serum/Plasma kit (Qiagen, Stanford CA) following manufacturer’s standard protocol.

### FACS Characterization of EVs

EVs were characterized by cytofluorimetric analysis using the following fluorescein isothiocyanate (FITC), allophycocyanin (APC) or phycoerythrin (PE) conjugated antibodies: CD63 (Cat.n. 130–100-160, Miltenyi Biotec, Germany), PDGFRβ (Cat.n. 130–105-280, Miltenyi Biotec, Germany), CD73 (Cat. n. 130–095-182, Miltenyi Biotec, Germany), αSMA (Cat. n. C6198, Invitrogen), CD44 (Cat. n. 130–095-195, Miltenyi Biotec, Germany), PDGFRα (Cat. n. LS-C107240, LSBio), FAP (ab207178, Abcam, Cambridge, United Kingdom) and fluorescent secondary Rabbit IgG antibody (A-11012, Thermo Fisher Scientific, Waltham, MA, USA). Conjugated mouse non-immune isotypic immunoglobulin G (IgG) (Miltenyi Biotec, Germany) was used as control. Briefly, 10 µl of EVs were labeled for 15 min at 4 °C with antibodies and immediately diluted 1:3 with saline solution and acquired [28]. Cytofluorimetric analysis were performed using the CytoFLEX flow cytometer (Beckman Coulter) with CytExpert software. Each analysis includes 3 biological replicates.

### Transmission electron microscopy analysis of EVs

EVs were analyzed using transmission electron microscopy analysis. For this, EV samples were placed on 200 mesh nickel formvar carbon-coated grids (Electron Microscopy Science, Hatfield, Pennsylvania, USA) and left to adhere for 20 min. Next, grids were incubated with 2.5% glutaraldehyde containing 2% sucrose. After washing in distilled water, samples were negatively stained with Nano-W and Nano-Van (Nanoprobes, Yaphank, New York, USA) and analyzed using a Jeol JEM 1010 electron microscope (Jeol, Tokyo, Japan) as described in [29].

### Proliferation assay

5 × 10^3^ cells/well were plated in 96 well plates in complete medium and starved for 24 h. Complete medium was then added and cells were allowed to grow for 1, 2, 3 and 5 days, fixed with 2.5% glutaraldehyde and stained with 0.1% crystal violet. The dye was solubilized using 10% acetic acid and optical density measured directly in plates using Promega GloMax®-Multi Detection System (Promega, Madison, WI) at 600 nm wavelength. For experiments with CM, tumor cells were pretreated for 24 h with CM from the different stroma cells, then complete medium was added and the assay performed as described above.

### Transwell migration, Transendothelial migration and Wound healing assays

To measure migration 3 × 10^5^ B16-F10 or 1 × 10^5^ EO771 cells or 1 × 10^5^ MEFs or CAFs were seeded in serum-free media in the upper chambers of cell culture inserts (Transwells) with 8.0 μm pore size membrane (24‐well format, Becton Dickinson, NJ). The lower chambers were filled with complete growth media. After 18–20 h, the migrated cells present on the lower side of the membrane were fixed in 2.5% glutaraldehyde, stained with 0.1% crystal violet and photographed using an Olympus IX70 microscope [19]. For transendothelial migration, 10^5^ HUVECs labelled with CellTracker™ Green CMFDA (Molecular Probes, Invitrogen Life Technologies) according to the manufacturer's instructions were seeded in complete medium in the upper part of transwell inserts with 5.0 μm pore size membrane (24-well format, Costar, Corning Incorporated, NY) coated by gelatin, and grown for 72 h, till confluency. Then, 3 × 10^5^ B16-F10 or 1 × 10^5^ EO771 cells were labelled with CellTracker™ Orange CMRA (Molecular Probes, Invitrogen Life Technologies), according to the manufacturer's instructions and seeded in HUVEC's complete medium onto the HUVEC–CMFDA monolayer on the upper side of the transwell. After 20 h, HUVECs and non-transmigrated cells were removed and the red-fluorescent (CMRA) cells that migrated on the lower side of the membrane were fixed in 4% paraformaldheyde and photographed using Zeiss AxioObserver microscope with ApoTome Module. Migration, invasion and transendothelial migration were evaluated by measuring the area occupied by migrated cells using the ImageJ software (http://rsbweb.nih.gov/ij/). For CM or EV experiments, cells were pretreated for 24 h with CM or EVs (5000 EVs/cell) before seeding. The wound healing motility assay was used to measure two dimensional movements. Cells were grown to confluency in six-well plates, serum starved or treated with CM or EVs (5000 EVs/cell) for 24 h, then a cross wound was made on the monolayer using a sterile 200 μl pipette tips. Cells were rinsed three times with Phosphate Buffered Saline (PBS) and placed in either serum-free DMEM or 10% FBS-DMEM. Two-dimensional cell movements were quantitated by measuring the distance covered by the migrating cells. For each experiment the four arms near the cross were photographed. Photos were taken at t = 0 h and at t = 6 h for B16-F10 cell or at t = 24 h for NIH3T3, HS5, EO771, MA-2 and 4175-TGL using a Zeiss AxioObserver microscope (Zeiss). Images were analyzed with ImageJ Software (http://rsbweb.nih.gov/ij/). The two-dimensional movement of the cells was quantitated by measuring the distance between the two edges of the wound and the formula described in [30] used to estimate cell speed.

### In vivo tumor and metastasis assays

For experimental metastasis assays, 5 × 10^5^ syngeneic B16-F10 (in 200 µL of PBS) were injected into the tail vein of 8–10 weeks old wild type (miR-214^wt^) or miR-214^over^ or miR-214^ko^ mice. Mice were dissected 8 days later and lung surface metastases counted in fresh total lungs using a Nikon SMZ1000 stereomicroscope (Nikon, Japan), then lungs were formalin-fixed, cut in small pieces, paraffin-embedded, sectioned and haematoxylin & eosin (H&E)-stained. Micrometastases were evaluated on specimens, with an Olympus BH2 microscope (Olympus, Japan). Spontaneous dissemination was evaluated in 8–10 weeks old wild type (miR-214^wt^) or miR-214^over^ or miR-214^ko^ mice subcutaneously injected with 5 × 10^5^ syngeneic B16-F10 or 5 × 10^5^ EO771 cells (in 200 µL of PBS). Mice were sacrified 45 days (B16-F10) or 30 days (EO771) after injections and tumors were harvested and weighed. For B16-F10 cells, subcutaneous tumors were surgically removed 15 days after injection. 30 days later, animals that were free of any local recurrence were further analyzed for the presence of Circulating Tumor Cells (CTCs), derived as described in [31]. Briefly, blood was collected by heart puncture with a 25G needle syringe in the presence of heparin. Blood was plated in tissue culture medium, and 3 days later tumor cells were washed, and then colonies or total number of cells were counted one week later. For some experimental groups, lungs were formalin-fixed, cut in small pieces, paraffin-embedded, sectioned and haematoxylin & eosin (H&E)-stained. Micrometastases were evaluated on specimens, with an Olympus BH2 microscope (Olympus, Japan). For Extracellular Vesicle (EV) treatments, 5 µg of EVs were administered (tail vein) to miR-214^ko^ mice 24 h before the tail vein injection of B16-F10 cells. For MA-2 experiments, subcutaneous tumors and experimental lung metastases were obtained in immunosuppressed mice as described in [11], primary tumors and metastases were dissected and RNA isolated.

### *In vivo* extravasation assay

Extravasation of B16-F10 cells was evaluated as described in Orso et al. [19]. Briefly, 1 × 10^5^ B16-F10 cells, previously labeled with CellTracker Orange CMRA (Molecular Probes, Invitrogen Life Technologies), were injected into the tail vein of 4- to 6-week-old miR-214^wt^, miR-214^over^ or miR-214^ko^ mice. 2 h or 48 h later, mice were sacrificed, lungs were dissected and photographed *in toto* using a Leica MZ16F fluorescence stereomicroscope and red fluorescence was quantified 48 h post- injection using the ImageJ software (http://rsbweb.nih.gov/ij/). Lungs were embedded in OCT (Killik, BioOptica), frozen, cryostat-cut in 6-μm-thick sections. Localization of tumor cells, inside/outside the vessels, was evaluated on sections at a Leica TCS SP8 confocal system (Leica Microsystems), following blood vessels staining with an anti-CD31 primary antibody in immunofluorescence. For Extracellular Vesicle (EV) treatment experiments, B16-F10 cells were pretreated for 24 h with 5,000 EVs/cell of the indicated sources before tail vein injections. Alternatively, 5ug of EVs were administered (tail vein) to miR-214^ko^ mice and subsequently tail vein injected with B16-F10 cells 24 h later. Relative extravasated cells were measured as fold change of extravasated cells in miR-214^over^ mice versus the values obtained in miR-214^wt^ or miR-214^ko^ animals. In detail, when miR-214^wt^ versus miR-214^over^ were compared, we performed the following calculation: we counted the number of extravasated cells in each miR-214^wt^ mouse and evaluated the average, we divided each figure by the average, we plotted the single values and showed the average as a line (equal to 1). Similarly, we counted the number of extravasated cells in each miR-214^over^ mouse and calculated the average, we divided each value by the average of extravasated cells in miR-214^wt^ animals and plotted the obtained results plus the average. A similar approach was used when miR-214^over^ versus miR-214^ko^ were analyzed.

### Stroma and IL-6/STAT3 signature correlation analysis in human tumors

TCGA data have been downloaded from the gdc portal (https://portal.gdc.cancer.gov/). Tumor purity data have been obtained from: http://genboree.org/theCommons/documents/569 (EDec), https://bioinformatics.mdanderson.org/estimate/disease.html (Stromal scores), and the TCGA biolinks R package (ABSOLUTE, IHC). TCGA samples corresponding to tumors (“Tumor metastatic” for SKCM and “Primary tumor” for BRCA) have been selected for the analyses and matched wih the corresponding stromal scores. The correlation between miR-214 expression and IL-6/STAT3 activity was inferred from the Pearson’s correlation between miR expression and a set of IL-6/STAT3 activity signatures’ expression. More in detail, for each TCGA sample having both mRNA and miR expression profiles, the sum of log transformed expression values of each IL-6/STAT3 signature’s genes was computed, and correlated with the corresponding log transformed miR expression value. AZARE_sig [32], DAUER_sig [33], IL-6_sig (from MSigDB, [34]), Alvarez_sig [35], TH_sig [36], stat3_sig [37], Jak/STAT (from MSigDB [34]). Analyses have been performed in R (version 3.5.1). Plots have been generated with pheatmap and ggplot2 R packages.

### Statistical analysis

The results are shown as mean ± Standard Deviation (SD) or ± Standard Error of Mean (SEM), as indicated. Each data group was first evaluated with Shapiro–Wilk normality test and, based on results, values were examined with parametric or non-parametric tools. *t*-test was used for parametric analyses between two groups. Instead, Mann–Whitney test was applied for non-parametric evaluations. When comparisons for more than two groups were performed, 1-way or 2-way ANOVA tests were chosen for parametric analyses, instead Kruskal–Wallis tests were applied for non-parametric evaluations. * = *P* ≤ 0.05; ** = *P* ≤ 0.01; *** = *P* ≤ 0.001 were considered to be statistically significant.

## Results

### miR-214 expression correlates with stroma components in human melanoma metastases and primary breast tumors and is highly expressed in stroma cells

**Fig. 1:**
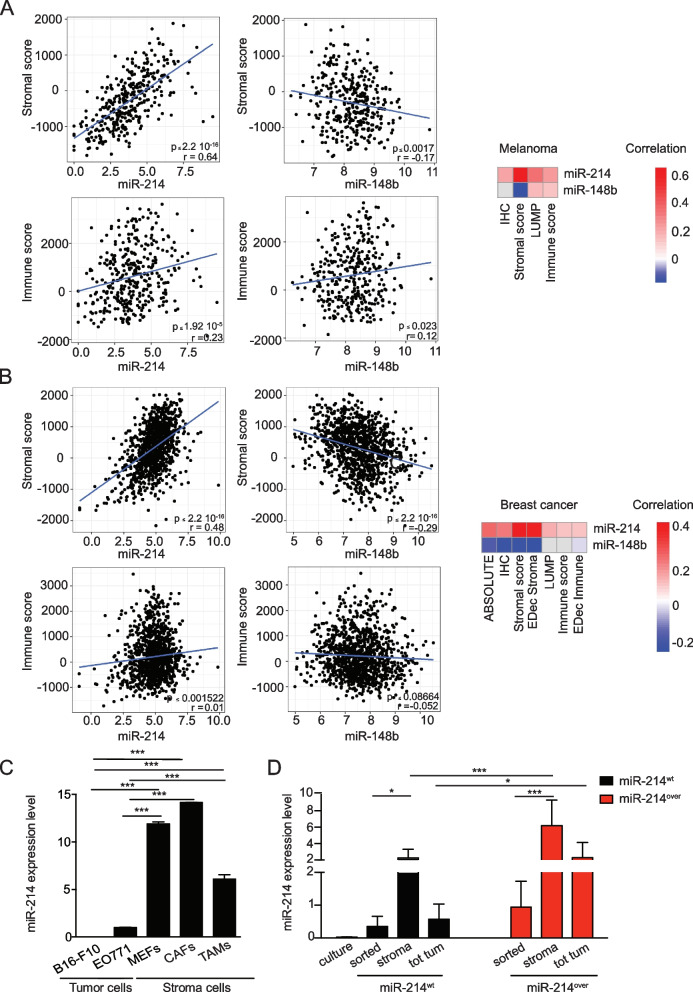
miR-214 expression correlates with stroma components in human tumors and it is highly expressed in stroma fibroblasts. **A-B** Significant correlations between miR-214 (positive) and miR-148b (negative) expression and stroma/immune cell composition in TCGA-SKCM (**A**) and TCGA-BRCA (**B**), for metastasis or primary samples, are presented as “scores”, based on 4 (**A**) or 7 (**B**) different evaluation methods, in plots and heatmaps (Red = positive; white/blue = negative; grey = no correlation). Non significant relationships: *p*-value > 0.05. Estimates: *EDec Immune/Stromal* = Epigenomic Deconvolution of stromal/immune percentage [38]; *Immune Score* = ssGSEA based on gene expression profiles of 141 immune genes [39]; *LUMP* = Leukocytes Unmethylation for Purity, which averages 44 non-methylated immune-specific CpG sites [40]; *Stromal score* = ssGSEA based on gene expression profiles of 141 stromal genes [39]; *IHC* = estimated by image analysis of haematoxylin and eosin stained slides produced by the Nationwide Children's Hospital Biospecimen Core Resource [41]; *ABSOLUTE* = based on somatic copy-number data [42]. **C** miR-214 expression levels in murine tumor, stroma and immune cells as indicated and evaluated by qRT-PCR analysis. **D** GFP^+^ B16-F10 cells were subcutaneously injected into miR-214^wt^ and miR-214^over^ syngenic mice to generate tumors. 15 days later, mice were sacrificed, transplants dissected and tumor/stroma cells separated by FACS sorting. miR-214 expression levels were measured in GFP.^+^ B16-F10 cells before the injection (culture) or FACS sorted from subcutaneous tumors (sorted), in the remaining stroma cells, after the sorting (stroma) and in the total tumor mass (tot tumor) by qRT-PCR analysis, 3 animals per group (*n* = 3). **C-D** Results are represented as fold changes (mean ± SD of triplicates) relative to the median, normalized on U6. Two independent experiments were performed and representative results are shown. MEFs = murine embryo fibroblasts; CAFs = cancer associated fibroblasts; TAMs = Tumor Associated Macrophages; SD = standard deviation. H&E = Haematoxylin & Eosin; *P ≤ 0.05; **P ≤ 0.01; ***P ≤ 0.001

Based on the evidence of miR-214 in promoting cancer dissemination [11] and on the increasing information underlying the essential role of the TME in cancer progression, the distribution of miR-214, and its downstream player, the anti-metastatic miR-148b [12, 19] was evaluated within metastases or primary tumor masses of human melanomas and breast cancers within the TCGA database. Tumor purity of bulk samples, based on tumor and stroma components, was determined using several algorithms, considering gene expression, copy number alterations or epigenetic profiles [38–42]. From these analyses, miR-214 positively correlated (*p*-value < 0.05) with stroma and immune constituents for four out of five estimates in melanomas (Fig. 1A and S1A) and for all estimates in breast tumors (Fig. 1B and S1B). Instead, miR-148b negatively correlated (*p*-value < 0.05) with the same estimates for the “Stromal Score” approach [39] in melanomas and for four out of seven tested methods in breast tumors (Fig. 1A-B and S1A-B). Based on this evidence miR-214 expression in different murine or human stroma components was evaluated and compared with tumor cells. In particular, we analyzed Mouse Embryo Fibroblasts (MEFs), Cancer Associated Fibroblasts (CAFs), Tumor Associated Macrophages (TAMs) *versus* B16-F10 mouse melanoma and EO771 tumor mammary gland cells (Fig. 1C). Furthermore, we evaluated miR-214 expression in: GFP^+^ B16-F10 cells in “culture”; whole subcutaneous tumors grown in miR-214^wt^ and miR-214^over^ syngeneic mice (see below), indicated as “tot tumor”; GFP + cells derived from xenotransplants following FACS sorting, marked as “sorted” tumor cells or the remaining “stroma” components (Fig. 1D). In all analyses, miR-214 was found more elevated in stroma than in tumor components. The same investigations were carried out in human Mesenchymal Stem Cells (hMSCs) and in HS5 bone marrow stroma cells *versus* human melanoma MA-2 or breast cancer 4175-TGL cells and similar results were observed (Fig. S1C-D). In addition, miR-214 levels significantly increased in xenografts derived from B16-F10 and MA-2 melanoma cells or in dissected lung metastases compared with tumor cells kept in culture (Fig. S1E-F). Moreover, increased levels of miR-214 were observed in B16-F10 and MA-2 cells co-cultured with MEFs or hMSCs or when B16-F10 cells were treated with hMSC-derived Conditioned Medium (CM) for 24 h (Fig. S1G-H). This evidence suggests a high production or storage of miR-214 in stroma cells, in particular for MEFs, CAFs and mesenchymal cells (MSCs and HS5), and a possible crosstalk between tumor and stroma cells involving miR-214.

### Stroma miR-214 promotes metastasis formation

**Fig. 2:**
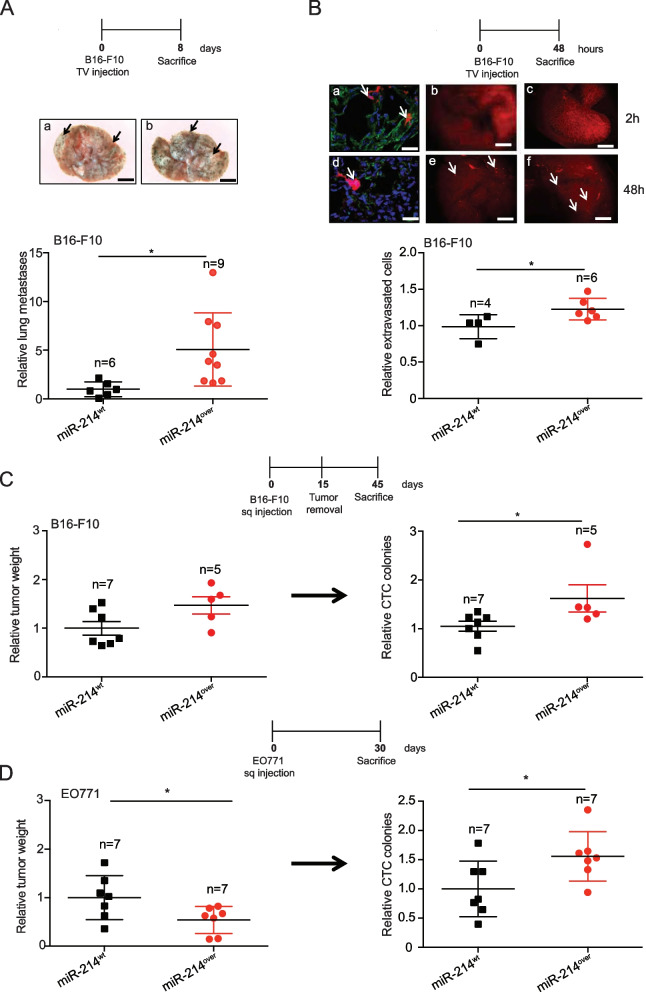
Overexpression of miR-214 in stroma cells favors metastasis formation of melanoma and breast cancer cells. **A** Analyses of experimental lung metastasis formation 8 days after tail vein injection of B16-F10 cells into miR-214^wt^ and miR-214^over^ syngeneic mice. Representative pictures of the whole lungs (a, b; scale bar = 2 mm), arrows indicate metastasis formations. Relative lung metastases between the two groups of mice is shown in the graph for the indicated number (n) of animals. **B** CMRA-labeled B16-F10 cells (red) were injected into the tail vein of miR-214^wt^ and miR-214^over^ syngeneic mice and extravasation was evaluated 2 h (a, b, c) or 48 h (d, e, f) later. Representative fields of murine lung sections stained for CD31 (green) to highlight blood vessels and counterstained with DAPI (blue); scale bar: 25 µm (a, d). Representative pictures of whole lungs containing red-labeled B16-F10 cells are shown; scale bar: 1 mm (b, c, e, f). Relative extravasated cells in the whole lungs at 48 h is shown in the graph for the indicated number (n) of mice, as mean ± SEM. **C-D** In vivo tumor growth and metastatic dissemination of B16-F10 (**C**) or EO771 (**D**) cells injected subcutaneously in miR-214^wt^ and miR-214.^over^ syngeneic mice, 45 or 30 days pos-inoculation respectively. In (**C**) tumors were removed 15 days post-injection and analyzed. Relative primary tumor weight and Circulating Tumor Cells (CTCs) are shown in the graphs as mean ± SEM, for the indicated number of mice (n). Two independent experiments were performed and representative results are shown. CMRA = CellTracker™ Orange; CD31 = cluster of differentiation 31; *n* = number of mice; DAPI = 4’, 6-diamidino-2-fenylindole SEM = standard error of mean. *P ≤ 0.05; **P ≤ 0.01; ***P ≤ 0.001

When exploring the potential contribution of stroma miR-214 in tumor progression coordination, we generated a total body miR-214 overexpression (miR-214^over^) mouse model, based on the Cre-loxP recombination system [43], and on the intervention of a Balancer-Cre mouse [16], to use as recipient for tumor cells (Fig. S2A). Mice were viable and fertile and did not show any developmental defect (data not shown). Increased miR-214 expression was confirmed in whole miR-214^over^ embryos compared to wild type (miR-214^wt^) controls (E12.5) by qRT-PCR (Fig. S2B), Northern Blot (Fig. S2C) and in situ hybridization (Fig. S2D) analyses. When B16-F10 melanoma cells were injected in the tail vein of miR-214^over^ (*n* = 9) or miR-214^wt^ (*n* = 6) syngeneic mice and lung metastatic nodules were evaluated 8 days later, increased dissemination was observed for miR-214^over^ mice as shown in Fig. 2A (graph and a, b images), thus suggesting that stroma miR-214 influenced the ability of tumor cells to seed in the lungs. In order to specifically evaluate the contribution of stroma miR-214 to extravasation, CMRA-labeled B16-F10 cells (red) were tail vein injected and extravasation was measured in the lungs 48 hours (h) later. Increased ability of tumor cells to cross the vessels was evidenced in miR-214^over^ mice compared to miR-214^wt^ animals, as illustrated in Fig. 2B (graph and images). This was not the consequence of a different lodging in the lung microvasculature since the same number of cells was found in the lungs of miR-214^over^ and miR-214^wt^ mice 2 h post-injection (Fig. 2B: b, c *versus* e, f). To note that most of the cells (red) were found inside the vessels at 2 h and in the lung parenchima at 48 h (Fig. 2B a, d), as demonstrated by CD31 (green) staining for the endothelial cells and DAPI (blue) counterstaining for DNA. The increased ability of tumor cells to disseminate in syngeneic miR-214^over^ mice was further observed when spontaneous metastasis formation experiments were performed. Here, B16-F10 melanoma or EO771 mammary tumor cells were subcutaneously injected in miR-214^over^ or miR-214^wt^ mice and relative Circulating Tumor Cell (CTC) colonies (Fig. 2C-D) or spontaneous lung metastases (Fig. S3A-B) were evaluated 45 or 30 days post-injection. It is important to note that B16-F10 tumors were removed 15 days post-injection to promote tumor dissemination. No increase in primary tumor growth was seen 15 or 30 days post-injection, shown as relative tumor weight, suggesting that stroma miR-214 favors tumor cell dissemination but not tumor growth (Fig. 2C-D). In fact, for EO771 we even observed a slight decrease in tumor weight. It can therefore be concluded from all these experiments that stroma miR-214 supports tumor cell dissemination.

### miR-214 derived from stroma EVs influences metastatic traits of tumor cells

**Fig. 3:**
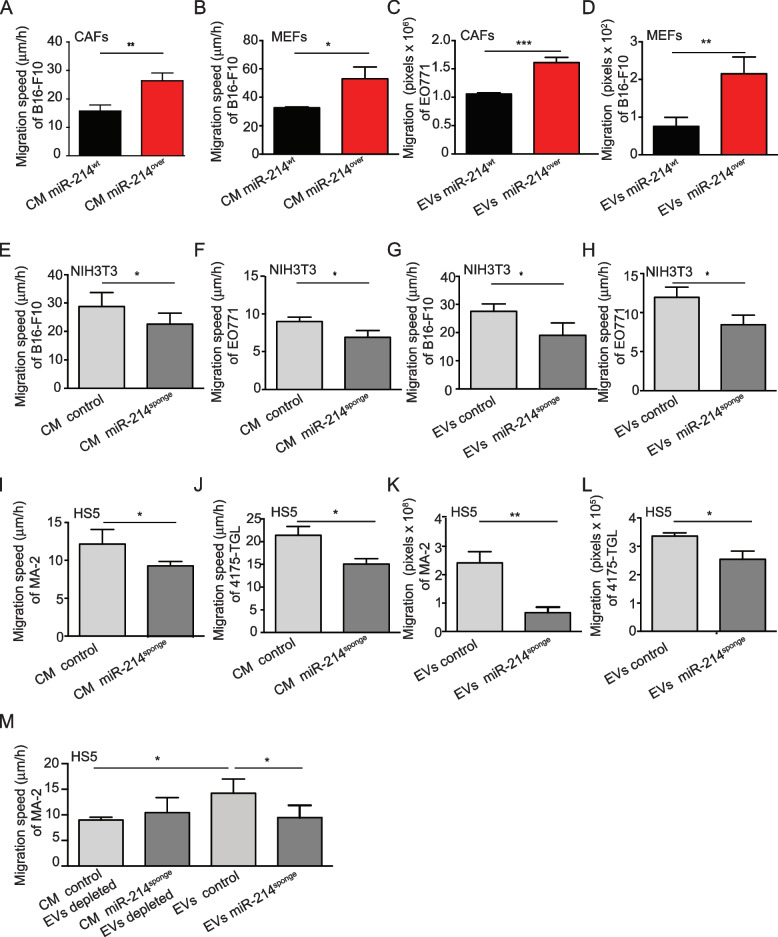
Stroma components favor tumor cell motility in a miR-214-dependent manner. **A**–**L** Wound healing (**A**, **B**, **E**, **F**, **G**, **H**, **I**, **J**) or transwell (**C**, **D**, **K**, **L**) assays for mouse B16-F10 or EO771 or human MA-2 or 4175-TGL cells were pretreated for 24 h with conditioned medium (CM) or Extracellular Vesicles (EVs) derived from either miR-214^wt^ and miR-214^over^ CAFs (**A**, **C**) or MEFs (**B**, **D**) or, from either NIH3T3 mouse fibroblasts (**E**-**H**) or HS5 human bone marrow stroma cells (**I**-**L**) previously transduced with a miR-214sponge (miR-214^sponge^) or an empty (control) expressing vector. **M** Wound healing assay for MA-2 cells pretreated for 24 h with EV-depleted Conditioned Medium (CM) or EVs derived from HS5 cells previously transduced with a miR-214sponge (miR-214^sponge^) or an empty (control) expressing vector. All transwell migration assays were evaluated 18 h later; while wound healing motility assays were evaluated 6 h (B16-F10)—18 h (EO771, MA-2)—24 h (4175-TGL) later. All results are shown as mean ± SEM and respectively shown as mean ± SEM of the area (pixels) or distance/time (μm/h of 10 pictures/duplicates) covered by migrated cells. For (**A**, **B**, **E**, **F**, **G**, **H**; **I**, **J**, **M**) experiments were performed at least twice as duplicates and pooled results of two or three experiments are shown. For (**C**, **D**, **K**, **L**) experiments were performed as triplicates and representative experiments are shown. CAFs = cancer associated fibroblasts; MEFs = murine embryo fibroblasts; SEM = standard error of mean. *P ≤ 0.05; **P ≤ 0.01; ***P ≤ 0.001

**Fig. 4:**
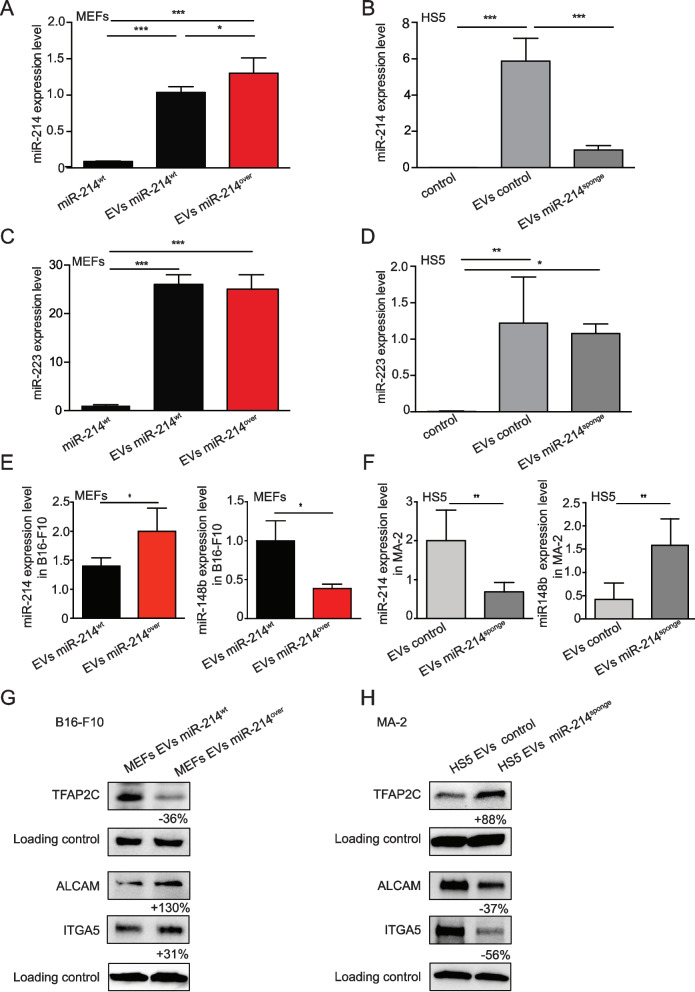
miR-214 is transferred from stroma to tumor cells. **A**-**D** miR-214 and miR-223 expression levels in Extracellular Vesicles (EVs) derived from miR-214^over^ and miR-214^wt^ MEFs or from HS5 were previously transduced with a miR-214sponge (miR-214^sponge^) or an empty (control) expressing vector, measured by qRT-PCR analysis. **E**–**F** miR-214 expression levels in B16-F10 and MA-2 cells pretreated for 24 h with EVs derived from miR-214^over^ or miR-214^wt^ MEFs or from miR-214 sponged HS5 (miR-214^sponge^) or empty controls (control), measured by qRT-PCR analysis. **A-F** Results are shown as fold changes (mean ± SD of triplicates) relative to controls, normalized on U6. At least 2 independent experiments (with triplicates) were performed and representative results are shown. **G-H** Expression levels of TFAP2C, ALCAM and ITGA5 in B16-F10 (**G**) and MA-2 (**H**) cells pretreated for 24/48 h with EVs derived from miR-214^over^ and miR-214^wt^ MEFs (**G**) or from HS5 (**H**) miR-214sponged HS5 (miR-214^sponge^) or empty controls (control), measured by Western Blot analysis. Protein modulations were calculated relative to controls, normalized on the loading control and expressed as percentages. MEFs = murine embryo fibroblasts; SEM = standard error of mean. SD = standard deviation. *P ≤ 0.05; **P ≤ 0.01; ***P ≤ 0.001

The influence of various miR-214-rich stroma cells on tumor cell metastatic traits in vitro was evaluated with the aim of dissecting the cellular and molecular mechanisms involved in the control of tumor cell dissemination by stroma miR-214. For this purpose, Murine Embryo Fibroblasts (MEFs) were derived from miR-214^over^ animals. Instead, Cancer Associated Fibroblasts (CAFs) were obtained from mammary tumors grown in MMTV-PyMT [17] mice crossbred with miR-214^over^ animals. Significant miR-214 overexpression was observed in both models (Fig. S4A, B). Conditioned medium (CM) or Extracellular Vesicles (EVs) derived from miR-214^over^ MEFs and CAFs or miR-214-depleted (miR-214^sponge^) [11] NIH3T3 and HS5 cells were placed in contact with mouse B16-F10 or EO771 or human MA-2 or 4175-TGL tumor cells and cell migration was evaluated by wound healing or transwell assays (Fig. 3). Characterization of stroma cells can be seen in Fig. S4C-H and S5A-F. No difference in cell morphology, N- or E-cadherin or α-SMA expression and proliferation was observed between miR-214^over^ or miR-214^wt^ CAFs (Fig. S4C-E). However, proliferation was more pronounced in miR-214^over^ MEFs (Fig. S4F) and increased migration was found for miR-214^over^ CAFs and MEFs (Fig. S4G-H). A strong decrease in miR-214 was observed in miR-214^sponge^ NIH3T3 and HS5 cells by qRT-PCR analysis (Fig. S5A-B), nevertheless, no dissimilarity in proliferation or migration was displayed between miR-214^sponge^ or control cells (Fig. S5C-F). When B16-F10 cells were treated with CM or EVs derived from miR-214^over^ CAFs and MEFs for 24 h, an increased migration and transendothelial migration was observed compared to controls (Fig. 3A-D, Fig. S6A-B). Conversely, treatment of B16-F10, EO771, MA-2 or 4175-TGL cells with CM or EVs derived from miR-214^sponge^ NIH3T3 or HS5 for 24 h led to a reduced migration compared to controls (Fig. 3E-L). Proliferation of tumor cells was not affected by treatments with CM derived from miR-214 overexpressing or sponged stroma cells (Fig. S7A-F). Interestingly, when the CM derived from miR-214^sponge^ HS5 cells was depleted of EVs and used to treat MA-2 cells no difference in migration was observed compared to controls (Fig. 3M) suggesting the importance of miR-214 in EVs. Likewise, when miR-214 content was measured in EVs derived from miR-214^over^ MEFs and miR-214^sponge^ HS5 cells by qRT-PCR analysis, increased and decreased levels were respectively detected compared to control EVs (Fig. 4A-B); while no alteration was observed in the same EVs for a control small non-coding RNA, miR-223 (Fig. 4C-D). Characterization of number, size, markers and morphology (transmission electron microscopy) of the various EVs used derived from MEFs, CAFs, NIH3T3 and HS5 did not reveal any difference as shown in Fig. S8A-M. All these data prompted us to hypothesize a transfer of miR-214 from stroma to tumor cells via EVs. Subsequently, we measured the levels of miR-214 and its downstream player, the anti-metastatic miR-148b, in tumor cells following CM or EV treatments. As shown in Fig. 4E-F, Fig. S9A-H, S10A-C modulated levels of miR-214 and miR-148b have been detected in various tumor cells. Precisely, increased miR-214 and decreased miR-148b expression were observed following treatments with CM or EVs derived from miR-214^over^ stroma cell while the opposite was observed for treatments with CM or EVs derived from miR-214^sponge^ stroma cells. Likewise, some direct targets of miR-214 or miR-148b involved in the coordination of metastases, turned out to be modulated (Fig. 4G-H, Fig. S10D-F). For instance, the expression of the AP-2γ transcription factor (TFAP2C), a miR-214 direct target, was found reduced by Western Blot (WB) analysis (Fig. 4G) following tumor cell treatments with miR-214^over^ EVs. Instead, expression of two adhesion molecules, the integrin alpha 5 (ITGA5) and the activated leukocyte cell adhesion molecule (ALCAM), both miR-148b direct targets, were increased (Fig. 4G). Opposite results were seen when tumor cells were treated with miR-214^ko^ or miR-214^sponge^ EVs (Fig. 4H, Fig. S10D-F). All these data suggest that stroma cells influence metastatic traits by transferring miR-214 from the microenvironment to tumor cells, which, in turn, modulates the expression of miR-214 or miR-148b direct targets.

### Depletion of stroma-derived miR-214 impairs tumor dissemination in vivo while its restoration promotes it

**Fig. 5:**
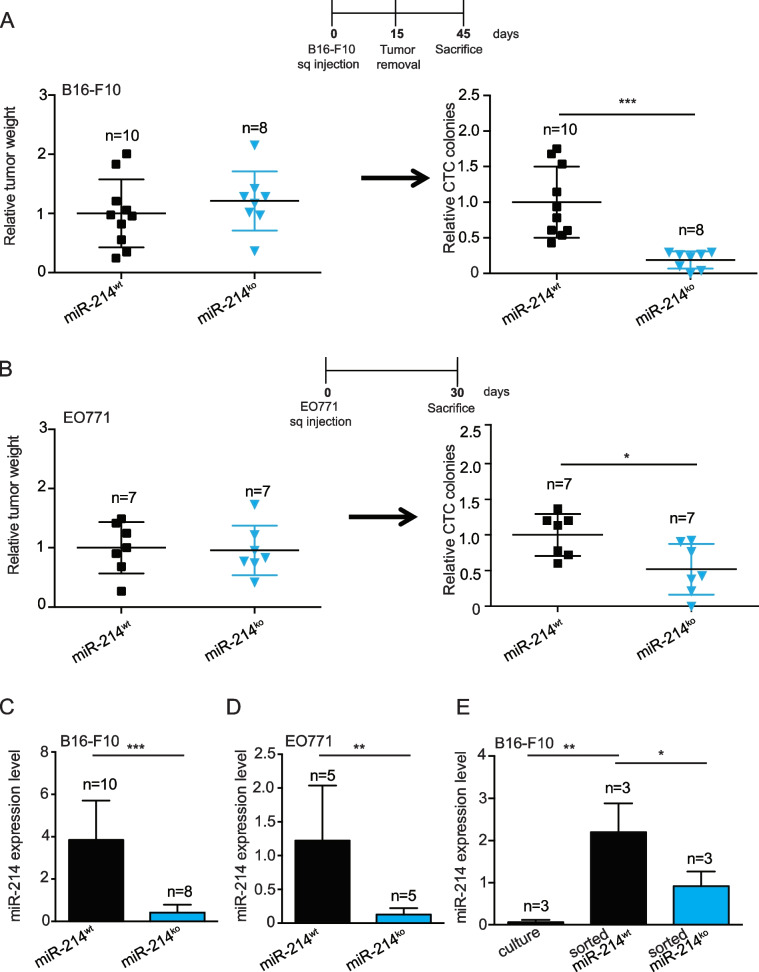
Ablation of miR-214 in stroma cells inhibits metastasis formation of melanoma and breast cancer cells. **A-B** In vivo tumor growth and metastatic dissemination of B16-F10 (**A**) and EO771 (**B**) cells injected subcutaneously in miR-214^wt^ and miR-214^ko^ syngeneic mice, respectively 45 or 30 days post-inoculation. In (**A**) tumors were removed 15 days post-injection. Relative primary tumor weight (measured in grams) and number of Circulating Tumor Cells (CTCs) is shown in the graphs as mean ± SEM, for the indicated number of mice (n). **C-D** miR-214 expression levels assessed in B16-F10- and EO771-derived xenografts grown in miR-214^wt^ and miR-214^ko^ mice by qRT-PCR analysis for the indicated number (n) of animals. **E** GFP^+^ B16-F10 cells were subcutaneously injected into miR-214^wt^ and miR-214^ko^ syngenic mice to generate tumors. 15 days later, mice were sacrificed, transplants dissected and tumor/stroma cells separated by FACS sorting. miR-214 expression levels were measured in GFP^+^ B16-F10 cells before injection (culture) or FACS sorted from subcutaneous tumors (sorted) by qRT-PCR analysis, for the indicated number (n) of animals. **C-E** Results are shown as fold changes (mean ± SD of triplicates) relative to the median, normalized on U6. **E** Results are pools of 3 animals per group. n = number of mice. SD = standard deviation. SEM = standard error of mean. *P ≤ 0.05; **P ≤ 0.01; ***P ≤ 0.001

**Fig. 6:**
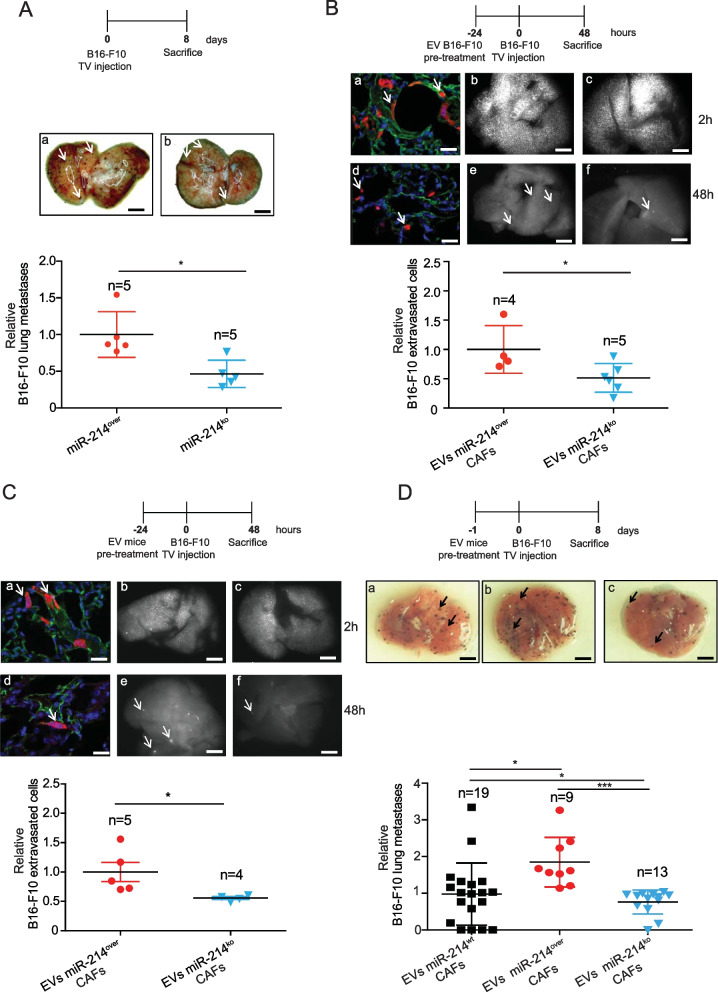
miR-214-rich Extracellular Vesicles (EVs) promote melanoma cell dissemination. **A** Analyses of experimental lung metastasis formation 8 days after tail vein injection of B16-F10 cells into miR-214^over^ and miR-214^ko^ syngeneic mice. Representative pictures of the whole lungs are shown (a, b; scale bar: 2 mm); arrows indicate metastasis formation. Relative lung metastases between the two groups of animals is presented in the graph for the indicated number (n) of mice. **B** CMRA-labeled B16-F10 cells (red) were pretreated for 24 h with EVs derived from miR-214^over^ or miR-214^ko^ CAFs and then injected into the tail vein of miR-214^ko^ syngeneic mice for the evaluation of extravasation 2 h (a, b, c) or 48 h (d, e, f) later. Alternatively **(C)**, extravasation was similarly evaluated for the same CMRA-labeled B16-F10 cells (red) injected into the tail vein of miR-214^ko^ syngeneic mice in which EVs derived from miR-214^over^ or miR-214^ko^ CAFs were administered 24 h earlier. For (**B**-**C**) Representative fields of murine lung sections stained for CD31 (green) to highlight blood vessels and counterstained with DAPI (blue) are shown in (a, d); scale bar: 25 µm. Representative pictures of whole lungs containing red-labeled B16-F10 cells are shown in (b, c, e, f); scale bar: 1 mm; arrows indicate extravasated cells. Relative extravasated cells in the whole lungs at 48 h is shown in the graph for the indicated number (n) of mice, as mean ± SEM. **D** Analyses of experimental lung metastasis formation 8 days after tail vein injection of B16-F10 cells into miR-214^ko^ syngeneic mice in which EVs derived from miR-214^wt^ or miR-214^over^ or miR-214.^ko^ CAFs were systemically administered 24 h earlier. Representative pictures of the whole lungs are shown (a, b, c); scale bar: 2 mm; arrows indicate metastasis formations. Relative lung metastases between the three groups of animals are presented in the graphs for the indicated number (n) of mice. CAFs = cancer associated fibroblasts; CD31 = cluster of differentiation 31; *n* = number of mice; DAPI = 4’, 6-diamidino-2-fenylindole; SEM = standard error of mean. *P ≤ 0.05; **P ≤ 0.01; ***P ≤ 0.001

To assess the effect of stroma miR-214 in in vivo dissemination, B16-F10 or EO771 cells were injected subcutaneously in the flank of miR-214^ko^ [18] or miR-214^wt^ syngeneic mice (*n* = 10/8 or *n* = 7 per group) and Circulating Tumor Cells (CTCs) or lung metastases were evaluated as normalized CTC colonies or relative lung metastases 45 or 30 days post-injection (Fig. 5 and Fig. S11A-B). To note that B16-F10-derived xenotransplants were removed 15 days post-injection to favor dissemination. While no difference in primary tumor growth was observed 15 (Fig. 5A) or 30 (Fig. 5B and S11A) days after injection, significant reduction of dissemination was evidenced for cells injected in miR-214^ko^ mice compared to controls (Fig. 5A-B and S11A-B), suggesting the relevance of stroma miR-214 in tumor progression. Noteworthy, almost undetectable or reduced levels of miR-214 were found in B16-F10 and EO771 tumors grown in miR-214^ko^ mice compared to controls, respectively (Fig. 5C-D). Likewise, decreased miR-214 expression levels were observed in B16-F10 cells sorted from tumors grown in miR-214^ko^ mice compared to tumors grown in miR-214^wt^ animals, evidencing, once again, the transfer of miR-214 from stroma to tumor cells in vivo (Fig. 5E). The significance of stroma miR-214 was further investigated by comparing lung dissemination for B16-F10 cells in miR-214-enriched (miR-214^over^) or miR-214-depleted (miR-214^ko^) mice (*n* = 4/5 per group), 8 days after tail vein injection (Fig. 6A). Here, an important decrease in tumor spread was observed in miR-214^ko^ mice measured for lung metastatic nodule formation (graph) relative to control mice and shown in representative whole lung pictures (a, b). Since dissemination of tail vein inoculated tumor cells depends on extravasation, crossing of the vessels was measured in miR-214^ko^ mice for the same B16-F10 cells previously treated, for 24 h, with EVs derived from miR-214^over^ or miR-214^ko^ CAFs, 48 h post-injection (*n* = 4/5). As shown in Fig. 6B (graph), an increased extravasation was observed for B16-F10 cells pretreated with miR-214-rich EVs compared to controls. Representative pictures of sections or whole lungs are also shown at 2 h or 48 h post-injection (a-f). To note that no difference in lodging was found for the two groups of cells, 2 h post-injection (Fig. 6B b, c). Tumor cells (red) were found inside the vessels at 2 h whereas, in the lung parenchima at 48 h (arrows), as demonstrated by CD31 (green) staining for the endothelial cells and DAPI (blue) counterstaining for DNA (Fig. 6B a, d). In vitro transendothelial migration experiments led to similar results. In fact, as shown in Fig. S12A, migration of B16-F10 cells through a HUVECs monolayer on top of a porous membrane was increased in cells pretreated for 24 h with EVs derived from miR-214^over^ CAFs compared to miR-214^wt^ or miR-214^ko^ EV treatment; while an impairment in migration ability of B16-F10 cells could be appreciated in cells pretreated with miR-214^ko^ EVs compared to miR-214^wt^ EV-treated cells. In line, as shown in Fig. 6C, we observed that when miR-214^ko^ mice were inoculated with EVs derived from miR-214^over^, 24 h before B16-F10 cell injection, extravasation was more pronounced compared to mice inoculated with EVs derived from miR-214^ko^. More importantly, when miR-214^ko^ mice were inoculated with EVs derived from miR-214^over^, 24 h before B16-F10 cell injection, lung nodule formation was more pronounced compared to control mice inoculated with either miR-214^wt^ or miR-214^ko^-derived EVs (Fig. 6D graph and images). miR-214 levels were also measured in mouse blood samples 15 min post-delivery of different EVs, by qRT-PCR analyses. As shown in Figure S12B, miR-214 expression was higher in mice injected with miR-214^over^ EVs compared to mice that received miR-214^wt^ or miR-214^ko^ EVs. Overall, our results reveal the essential role of stroma miR-214 in promoting tumor dissemination and suggest potential therapeutic interventions to target miR-214 not only in tumor cells but also in the stroma components.

### Tumor cells induce miR-214 accumulation in the stroma components of the TME

**Fig. 7:**
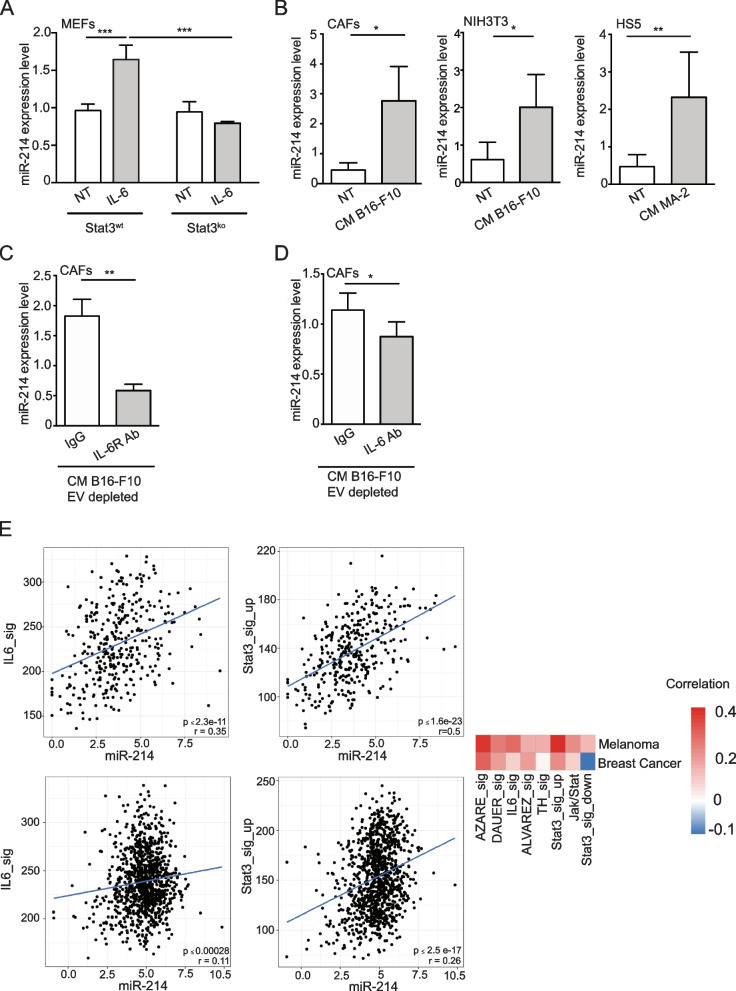
Tumor cells influence miR-214 expression in stroma cells. **A** miR-214 expression levels assessed by qRT-PCR analysis in Stat3^wt^ or Stat3.^ko^ MEFs, treated with IL-6 for 6 h. **B** miR-214 expression levels evaluated by qRT-PCR analysis in NIH3T3 or CAFs or HS5 cells following pretreatment with B16-F10 or MA-2 Conditioned Medium (CM) or left untreated (NT) for 6 h. **C-D** miR-214 expression assessed by qRT-PCR analysis in CAFs treated for 6 h with B16-F10 EV-depleted Conditioned Medium (CM) in presence of 50 μg/ml of anti-IL-6R Ab (**C**) or 10 μg/ml of anti-IL-6 Ab (**D**) or control IgG. **E** Significant positive correlations between miR-214 expression and different IL-6 and Stat3-related signatures in TCGA-SKCM (top) or TCGA-BRCA (bottom) datasets. Samples are presented in plots and heatmaps (Red = positive; white/blue = negative; grey = no correlation). Non-significant relationships: *p*-value > 0.05. *AZARE_sig* [32]; *DAUER_sig* [33]; *IL-6_sig* [34, 45] M5897; *ALVAREZ_sig* [35]; *TH_sig* [36]; *Stat3_sig_up* [37]; *Jak/Stat* [34, 45] M11564; *Stat3_sig_down* [36]. For (**A**-**D**) results are presented as fold changes (mean ± SD of triplicates) relative to controls, normalized on U6. At least 2 independent experiments (with triplicates) were performed and either representative results (**A**, **C**) or pools of all results (**B**, **D**) are shown. SD = standard deviation; Ab = antibody. *P ≤ 0.05; **P ≤ 0.01; ***P ≤ 0.001

**Fig. 8:**
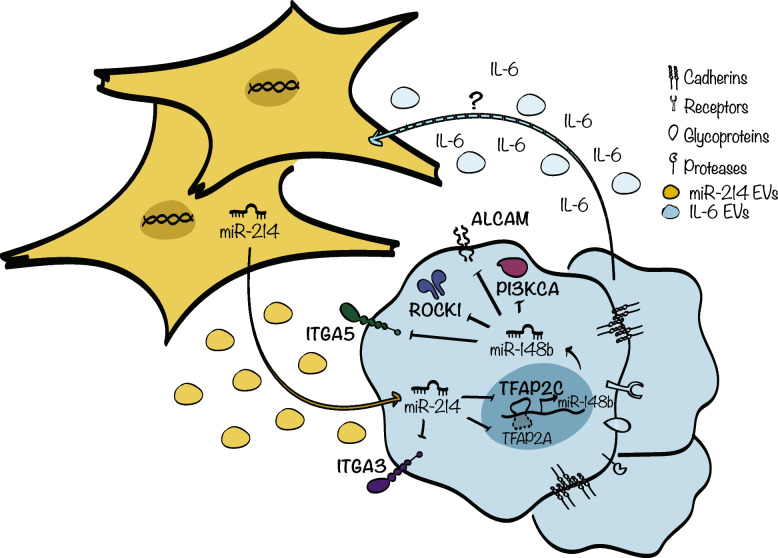
miR-214 in tumor-stroma cell interactions. Scheme illustrating miR-214-mediated crosstalk between tumor and stroma cells coordinating tumor dissemination

All data presented above reveal that stroma cells are rich in miR-214 and are able to transfer it to tumor cells via EVs, which, in turn, use it to activate their metastatic program. IL-6 signaling and its downstream player STAT3 are strongly involved in the crosstalk between cancer and stroma cells [44]. When we analyzed miR-214 expression, by qRT-PCR analysis, in STAT3^wt^ or STAT3^ko^ MEFs following IL-6 stimulation, increased levels of miR-214 in Stat3^wt^ but not Stat3^ko^ were observed, suggesting the importance of the IL-6/STAT3 pathway activation in miR-214 production (Fig. 7A). When CAFs, NIH3T3 and HS5 were treated with CM derived from B16-F10 or MA-2 cells for 6 h, miR-214 expression increased, compared to untreated (NT) controls, suggesting that tumor cells are able to induce miR-214 accumulation in stroma cells (Fig. 7B). However, when anti-IL-6 receptor (IL-6R Ab) or anti IL-6 (IL-6 Ab) blocking antibodies were added to EV-depleted CM derived from B16-F10 cells, reduced miR-214 induction was observed in CAFs, compared to control immunoglobulin (IgG) treatments (Fig. 7C-D), thus suggesting that IL-6 is secreted in tumor-stroma cell co-cultures and is used for the accumulation of miR-214 in stroma cells. To assess the relevance of miR-214/IL-6/STAT3 axis in human tumors, the correlation between miR-214 and IL-6 or STAT3 expression was evaluated in various signatures of melanoma and breast cancer samples and miR-214 resulted positively correlated (*p*-value < 0.05) with IL-6 and STAT3 expression in all the signatures analyzed [34, 36] as in Fig. 7E and Fig. S13A-B. Importantly, an anti-correlation was observed in melanoma and breast cancer samples when downregulated genes from an IL-6/STAT3 signature were used [32–37, 45] as in Fig. S13A-B. For the most part, our data suggest an important crosstalk between tumor and stroma cells involving the IL-6/STAT3/miR-214 axis and the release of miR-214-rich EVs, which, in turn, lead to the activation of a malignancy pathway, necessary for tumor dissemination, as summarized in Fig. 8.

## Discussion

The following relevant conclusions can be drawn from our work:

- miR-214 of stroma origin is essential for melanoma and breast cancer progression. In fact, increased tumor dissemination was observed when newly generated miR-214 overexpressing (miR-214^over^) transgenic mice were used as recipients for tumor cell injections. Conversely, tumor progression was impaired when cancer cells were introduced in miR-214 knock out mice (miR-214^ko^). Furthermore, we showed that miR-214 is released by stroma cells within Extracellular Vesicles (EVs) that are recovered by tumor cells and used to activate a metastatic program, which includes transcription factors, adhesion molecules as well as another small non-coding RNA, the anti-metastatic miR-148b. Many studies have demonstrated the importance of EVs in cancer progression by mainly focusing on the release of tumor cells versus stroma cells [9]. Here, we highlight the importance of EVs released from stroma cells for tumor malignancy. CM derived from miR-214-deprived stroma cells does not affect tumor cell migration when deprived of EVs, specifically suggesting the role of miR-214-rich EVs for tumor cell migration, extravasation, circulation in the blood stream and metastasis formation. Similarly, in physiological conditions, the relevance of miR-214-rich EVs has been evidenced in various organs. For instance, osteoclast-specific miR-214 knock-in mice have increased serum exosomal miR-214, which inhibits osteoblast activity and bone formation [46]. A similar situation has been observed in elderly women with fractures and in ovariectomized mice [47]. Bone Marrow Derived Mesenchymal Stem cells (BMDSCs) also release miR-214-rich exosomes used by cardiac stem cells to suppress oxidative stress injury through CaMKII silencing [48]. In non-small cell lung cancer, gefinitib resistance is propagated from resistant to non-resistant cells by exosomal miR-214 [49]. High levels of miR-214 have been found in serum of breast cancer patients and are linked to malignancy and metastatic spread to regional lymph nodes [50].
- miR-214 is strongly expressed in stroma and immune cells from the Tumor Micro Environment (TME) of human and mouse melanoma as well as breast cancer samples. In particular, we found it highly expressed in Cancer Associated Fibroblasts (CAFs) and Mesenchymal Stem Cells (MSCs), suggesting a potential role for the TME in miR-214 production and storage during tumor progression. Various miR modulations have been previously observed in fibroblasts in different types of tumor masses [51–53]. This is, however, the first study which demonstrates the significant increase of miR-214 in CAFs and its consequent release to tumor cells.
- Tumor cells are able to stimulate miR-214 expression in the stroma counterparts which is linked to IL-6 production and IL-6/STAT3 signaling. Increased miR-214 levels in Stat3-positive but not in Stat3^ko^ Mouse Embryo Fibroblasts (MEFs) following IL-6 stimulation were observed. Moreover, the treatment of B16-F10-derived CM with blocking antibodies for IL-6 or IL-6R impaired miR-214 production in CAFs, suggesting the relevance of this pathway for the production of miR-214 in the TME to generate a “miR-214 reservoir”. The high levels of miR-214 found in stroma cells could be linked to a more pronounced expression of STAT3/pSTAT3 and IL-6R. In fact, higher STAT3/pSTAT3 and IL-6R expression was found in CAFs compared to tumor cells (data not shown). The role of this pathway in tumor cells cannot, however, be ruled out. miR-214 expression correlates with IL-6 and STAT3 signatures in TCGA breast cancer and melanoma datasets. It is well known that inflammation promotes tumor progression mostly by inducing the release of growth factors and cytokines favoring the establishment of cancer stem cells as well as viability and the spread of tumor cells or disabling tumour-specific T cell functions [54]. Among the different inflammatory cytokines, IL-6 plays a primary role in the tumor-stroma cell crosstalk. Its dysregulation in many types of cancers and its elevated levels often correlate with worse prognosis in breast, ovarian, prostate and renal carcinomas, in melanomas as well as in multiple myelomas and lymphomas [55]. In particular, IL-6 has been proposed as a prognostic biomarker in patients with metastatic melanomas and it can be used to evaluate the efficacy of therapeutic treatments [55]. Once IL-6 binds to its receptor on the plasma membrane of tumor or stroma cells, the pathway JAK1/STAT3 is activated and controls genes involved in proliferation, survival, invasion and metastasis formation [56]. The role of miR-214 in inflammation has been observed in several non-neoplastic diseases. For instance, miR-214 exacerbates kidney damage and inflammation induced by hyperlipidemic pancreatitis [57], it mediates perivascular fibrosis in hypertension [58] and it is involved, together with IL-6/STAT3, in ulcerative colitis pathogenesis where its targeting reduces the severity of the disease [59, 60]. Instead, its role in tumor associated inflammation is poorly understood. However, our study reveals a critical role for the IL-6/STAT3/miR-214 axis during tumor progression which could be useful in clinical interventions. Indeed, therapeutic approaches targeting IL-6 signaling in neoplasia have been successful in preclinical settings [61, 62], but anti-IL-6 monotherapy in clinical trials for colorectal, ovarian and pancreatic cancers showed no beneficial outcome [63], possibly because of signaling alterations. For instance, in ovarian cancer, the administration of IL-6 neutralizing antibodies causes EGFR upregulation, while the combination of IL-6 neutralizing antibodies with Gefitinib, an EGFR inhibitor, promotes relevant anticancer activity [64]. Similarly, STAT3 displayed good potential in pre-clinical studies but not in clinical trials [65]. Our data set the basis for a combined therapy able to hit the IL-6/STAT3 pathway as well as miR-214.
- As we previously demonstrated, the inhibition of miR-214 in tumor cells and the systemic delivery of anti-miR-214 in mice bearing tumors strongly inhibits metastasis formation [19, 31]. However, in our previous work we performed a systemic targeting of miR-214 without any specificity for target cells. In our present study, we show that tumor cell dissemination is impaired by the absence of miR-214 in stroma cells, underlying the main role of stroma miR-214 in the malignancy process. We observed that when miR-214-null mice were injected with EVs derived from miR-214^over^ or miR-214^wt^ or miR-214^ko^ CAFs and subsequently with tumor cells, extravasation and lung metastasis formation were enhanced in mice treated with miR-214^over^ EVs compared to animals that received miR-214^wt^ or miR-214^ko^ EVs, suggesting that tumor cells need a reservoir of miR-214-rich EVs in order to disseminate. The analysis of the circulating miR-214 content, soon after inoculation of the different CAF-derived EVs in miR-214^ko^ mice, revealed increased miR-214 levels in mice injected with miR-214^over^ CAF-derived EVs compared to mice injected with EVs from miR-214^wt^ or miR-214^ko^ CAFs, thus proving the EV contribution to the increased miR-214 circulating levels. However, since we did not perform analyses at later time points, we cannot prove that miR-214 is directly taken up by tumor cells from EVs present in the blood stream. It is known that EVs are quickly up-taken and used by different tissue or tumor cells of the recipient organisms and eventually transferred [66, 67]. Future studies are necessary to carefully document if, where and when the passage of miR-214 from stroma EVs to tumor cells occurs in vivo. miR-214 overexpression has also a biological impact on stroma cells as shown by the fact that increased miR-214 expression in CAFs and MEFs does favor migration but is not essential. As a matter of fact, miR-214 depletion in fibroblasts or MSCs does not alter their migratory potential, further suggesting that stroma miR-214 is mostly used as “a reservoir” for tumor cells. Based on this evidence we aim to establish recommendations for therapeutic modalities which allow delivery of anti-miR-214 or miR-214 sponges not only to tumor cells but also to the stroma cells present in the TME.

## Conclusions

In conclusion, we demonstrate the ability of tumor cells to instruct cells of the TME to produce and store miR-214 which is subsequently released to tumor cells via EVs thus activating a metastatic process. We present strong genetic evidence that stroma miR-214 is essential for tumor dissemination underlying the relevance of specifically targeting stroma miR-214 to fight metastasis formation.

## Supplementary Information

**Additional file 1.**

## Data Availability

Data and material are available on request from the authors.

## Abbreviations

TME: Tumor Micro Environment
miRs: MicroRNAs
EVs: Extracellular Vesicles
CAFs: Cancer Associated Fibroblasts
MSCs: Mesenchymal Stem Cells
MEFs: Murine Embryo Fibroblasts
TAMs: Tumor Associated Macrophages
GFP: Green Fluorescent Protein
CM: Conditioned Medium
CMRA: CellTracker™ Orange
DAPI: 4’, 6-diamidino-2-fenylindole
CTCs: Circulating Tumor Cells
ALCAM: Activated leukocyte cell adhesion molecule
ITGA5: Integrin alpha 5
WB: Western Blot
IL-6R: Interleukin 6 Receptor
IgG: Immunoglobulin G
BMDMSCs: Bone Marrow Derived Mesenchymal Stem Cells
FLP: Flippase
DIG: Digoxigenin
SEM: Standard Error of the Mean
SD: Standard Deviation
