# Supplementary material for: Stroma-derived miR-214 coordinates tumor dissemination
Source: J Exp Clin Cancer Res. 2023 Jan 13;42:20. doi: 10.1186/s13046-022-02553-5 (PMC9837925; doi:10.1186/s13046-022-02553-5)
Supplement: Supplementary file 1 — Additional file 1. [file 13046_2022_2553_MOESM1_ESM.pdf]

SUPPLEMENTARY FIGURES

Figure S1

Expression of miR-214 in cells, tumors and metastases.

(A-B) Correlations between miR-214 and miR-148b expression and stroma/immune cell composition in TCGA-SKCM (A) and TCGA-BRCA (B), for metastasis or primary samples, are presented as "scores", based on different evaluation methods, in plots. Non-significant relationships: p-value >0.05. Estimates: *EDec Immune/Stromal* = Epigenomic Deconvolution of stromal/immune percentage (1); *LUMP* = Leukocytes Unmethylation for Purity, which averages 44 non-methylated immune-specific CpG sites (2); *IHC* = estimated by image analysis of haematoxylin and eosin stained slides produced by the Nationwide Children's Hospital Biospecimen Core Resource (3); *ABSOLUTE* = based on somatic copy-number data (4). **(C-H)** miR-214 expression levels were measured for the indicated cells, primary tumors or metastasis by qRT-PCR analysis. (**C-D**) Human tumor and stroma cells; **(E-F)** mouse B16-F10 or human MA-2 melanoma cells in culture or their derived total primary subcutaneous (sc) tumors or dissected metastases (mets) following sc or tail vein injections in syngeneic or immunosuppressed mice, respectively; (**G-H)** B16-F10 or MA-2 cells not treated (NT) or co-cultured with MEFs (G) or hMSCs (H) or treated with Conditioned Medium (CM) derived from hMSCs (H) for 24 h. Results are shown as fold changes (mean±SD of triplicates) relative to the median of miR-214 levels, normalized on U6 or U44 RNA. At least 2 independent experiments (with triplicates) were performed and either representative results (C, E, F) or pooled results of two independent experiments (D, G) are shown. MEFs=murine embryo fibroblasts; hMSCs=human mesenchymal stem cells; SD=standard deviation. *P≤0.05; **P≤0.01; ***P≤0.001.

Figure S2

Generation and characterization of the miR-214 overexpressing mouse model.

(A) Schematic overview of the strategy used to generate a conditional miR-214 overexpressing (miR-214^{over}) mouse model. The sequence of miR-214 precursor was cloned downstream of a constitutive active strong promoter (CAGGS) followed by a transcriptional STOP element (STOP) flanked by two LoxP sites (miR-214^{over} plasmid). This construct was specifically targeted in the 3' UTR of the Collagene alpha 1 (ColA1) genomic locus in previously engineered mouse Embryonic Stem Cells (mESCs) using the flippase (FLP) site-directed recombination technology. Recombinant ESCs were positively selected using hygromycin, driven by a PGK (phosphoglycerate kinase) promoter. (**B**) miR-214 expression levels were measured in miR-214^{wt} and miR-214^{over} total embryos (E12.5) by qRT-PCR analysis for the indicated number (n) of animals. (**C**) miR-214 expression levels were evaluated in miR-214^{wt} (n=5) and miR-214^{over} (n=3) total embryos (E12.5) by Northern Blot analysis using specific miR-214 and U6 small nucleolar RNA digossigenin-labeled LNA probes. (**D**) *In situ* hybridization analysis for miR-214 expression on Formalin-Fixed Paraffin Embedded (FFPE) miR-214^{wt} and miR-214^{over} total embryos (E12.5), using digossigenin-labeled LNA probes. Representative images of miR-214 expression in vertebrae (a, b) and liver (c, d) are shown; scale bar: 200 μm. **(B)** Results were calculated as fold changes (mean ± SD of triplicates) relative to the median of miR-214 levels for the indicated number (n) of embryos, normalized on U6 small nucleolar RNA levels. SD=standard deviation. *P≤0.05; **P≤0.01; ***P≤0.001.

Figure S3

***In vivo* tumor growth and metastatic dissemination.**

(A) *In vivo* tumor growth and metastatic dissemination of B16-F10 cells injected subcutaneously in miR-214^{wt} and miR-214^{over} syngeneic mice, 45 days post-inoculation. Tumors were removed 15 days post-injection. Relative primary tumor weight and relative number of lung metastases are presented as mean±SEM for the indicated number (n) of mice. Representative images of lung metastases are also shown. Arrows point to metastases. **(B)** Analysis of S100b expression in a lung lobe derived

from B16-F10-xenografts grown in miR-214^{wt} and miR-214^{over} mice (as in A) by qRT-PCR analysis. Results were calculated as fold changes (mean ± SD of triplicates) relative to the median of samples for the indicated number (n) of mice, normalized on 18S rRNA levels. SD=standard deviation. *P≤0.05; **P≤0.01; ***P≤0.001.

Figure S4

Characterization of miR-214-overexpressing stroma cells.

(**A-B)** miR-214 expression levels were measured in miR-214^{wt} and miR-214^{over}-derived CAFs (A) or MEFs (B) by qRT-PCR analysis. **(C)** Representative pictures of miR-214^{wt} or miR-214^{over} CAFs isolated from mice; scale bar: 150 μm. (**D)** Evaluation of N-cadherin, E-cadherin, α–SMA and loading control protein expression in different miR-214^{wt} or miR-214^{over} CAF preparations by Western Blot analysis. (**E-F)** Proliferation assays for miR-214^{wt} or miR-214^{over} CAFs (E) or MEFs (F). Results are indicated as mean ± SEM of proliferation rate, measured by Optical Density (OD) at 0h–72h (t0-t72). (**G-H)** Transwell Migration assays for miR-214^{wt} or miR-214^{over} CAFs (G) or MEFs (H). **(A, B)** Results were calculated as fold changes (mean ± SD of triplicates) relative to the median of miR-214 levels for the indicated number (n) of MEF and CAF preparations, normalized on U6 small nucleolar RNA levels. For (**E-H**) Results are shown as mean ± SEM of the area covered by migrated cells. All experiments were performed as triplicates and representative results are shown. CAFs=cancer associated fibroblasts; MEFs=murine embryo fibroblasts; SEM= standard error of mean; α–SMA=*alpha* Smooth Muscle Actin. *P≤0.05; **P≤0.01; ***P≤0.001.

Figure S5

Characterization of miR-214-sponged stromal cells.

(A-B) miR-214 expression levels assessed in NIH3T3 mouse fibroblasts (A) or HS5 human bone marrow stromal cells (B) previously transduced with a miR-214sponge (miR-214^{sponge}) or an empty (control) expressing vector by qRT-PCR analysis. Results are shown as fold changes (mean ± SD of

triplicates) relative to controls, normalized on U6. At least 2 independent experiments (with triplicates) were performed and representative results are shown. (**C-D)** Proliferation assays for the same NIH3T3 and HS5 used in (A-B). Results are indicated as mean ± SEM of the proliferation, measured by Optical Density (OD) at 0h–72h (t0-t72). **(E-F)** Wound healing assays for the same NIH3T3 and HS5 used in (A-B). Migration speed was evaluated after 6h (E) or 24h (F) as mean ± SEM of distance/time covered by migrated cells (μm/h of 10 pictures/duplicates). (**C-F**) All experiments were performed at least twice (with duplicates/triplicates) and representative results are shown. SEM= standard error of mean. SD=standard deviation. *P≤0.05; **P≤0.01; ***P≤0.001.

Fig. S6

Stroma miR-214 enhances transendothelial migration of tumor cells.

(A-B) Transendothelial migration through a HUVECs monolayer on top of a porous membrane for B16-F10 and EO771 cells pretreated for 24h with Extracellular Vesicles (EVs) derived from miR-214^{wt} and miR-214^{over} CAFs. Top: representative pictures of HUVECs monolayer before plating and B16-F10 and EO771 migrated cells. Bottom: results expressed as mean±SEM of the area (pixels) covered by migrated cells. 2 independent experiments (with triplicates) were performed and pooled results are shown. CAFs=cancer associated fibroblasts; SEM= standard error of mean. *P≤0.05; **P≤0.01; ***P≤0.001.

Fig. S7

Stroma miR-214 does not influence tumor cell proliferation.

(A-F) Proliferation assays for B16-F10 (A, C) or EO771 (B, D) or MA-2 (E) or 4175-TGL (F) cells pretreated for 24h with Conditioned Medium (CM) derived from either miR-214^{wt} or miR-214^{over} CAFs (A-B) or alternatively from NIH3T3 (C-D) or HS5 cells (E-F) previously transduced with a miR-214sponge (miR-214^{sponge}) or an empty (control) expressing vector. All results are presented as mean ± SEM of the proliferation rate, measured by Optical Density (OD) at 0h–72h (t0-t72). At least

two independent experiments as triplicates were performed, and representative results are shown. MEFs=murine embryo fibroblasts; CAFs=cancer associated fibroblasts; SEM= standard error of mean. *P≤0.05; **P≤0.01; ***P≤0.001.

Figure S8

Characterization of Extracellular Vesicles derived from stroma cells.

(A-H) Evaluation of number and size (nm) of Extracellular Vesicles (EVs) derived from miR-214^{wt}, miR-214^{over} or miR-214^{ko} MEFs and CAFs (A-D) or alternatively from NIH3T3 and HS5 (E-H) cells previously transduced with a miR-214sponge (miR-214^{sponge}) or an empty (control) expressing vector by Nanosight analysis. Values are shown as mean±SEM for the number (n) of indicated preparations. **(I-K)** Evaluation of EV and cell of origin markers (CD63, CD140a CD140b, CD73, CD44, α-SMA, Fap) in EVs derived from the indicated cells by FACS analysis shown as percentage (%) of signal intensity refereed to mean± SEM. **(L-M)** Electron microscopy images of EVs derived from miR-214^{wt}, miR-214^{over} or miR-214^{ko} CAFs (L) or alternatively from HS5 cells previously transduced with a miR-214sponge (miR-214^{sponge}) or an empty (control) expressing vector (M); scale bar: 100 nm. MEFs=murine embryo fibroblasts; CAFs=cancer associated fibroblasts; SEM= standard error of mean. *P≤0.05; **P≤0.01; ***P≤0.001.

Figure S9

miR-214 and miR-148b expression in tumor cells is modulated by stroma cell conditioned medium.

(A-H) miR-214 and miR-148b expression levels measured in mouse B16-F10 (A-C) and EO771 (B-D) or human MA-2 (E-G) or 4175-TGL (F-H) cells pre-treated for 24h with the Conditioned Medium (CM) derived from miR-214^{wt} and miR-214^{over} MEFs or alternatively from HS5 cells previously transduced with a miR-214sponge (miR-214^{sponge}) or an empty (control) expressing vector by qRT-PCR analysis. All results are shown as fold changes (mean±SD of triplicates) relative to the median

of samples, normalized on U6. At least 2 independent experiments (with triplicates) were performed and pooled results of two or three independent experiments are shown. MEFs=murine embryo fibroblasts; SD=standard deviation. *P≤0.05; **P≤0.01; ***P≤0.001.

Figure S10

The transfer of stroma miR-214 to tumor cells affects a pro-metastatic pathway.

(A-C) miR-214 or miR-148b expression levels in EO771 (A) and 4175-TGL (B, C) cells pre-treated for 24-48h with Extracellular Vesicles (EVs) derived from NIH3T3 (A) or HS5 (B, C) previously transduced with a miR-214sponge (miR-214^{sponge}) or an empty (control) expressing vector by qRT-PCR analysis. All results are shown as fold changes (mean±SD of triplicates) relative to the median of samples, normalized on U6. At least 2 independent experiments (with triplicates) were performed and pooled results of two or three independent experiments are shown. **(D-F)** TFAP2C, ALCAM and ITGA5 protein expression in B16-F10 (D), EO771 (E) and 4175-TGL (F) pre-treated for 24-48h with Extracellular Vesicles (EVs) derived from miR-214^{over} or miR-214^{ko} CAFs or alternatively from NIH3T3 or HS5 cells previously transduced with a miR-214sponge (miR-214^{sponge}) or an empty (control) expressing vector. Protein modulations were calculated relative to the indicated controls, normalized on the loading control and expressed as percentages. CAFs=cancer associated fibroblasts; SD=standard deviation *P≤0.05; **P≤0.01; ***P≤0.001.

Figure S11

Metastasis formation in a miR-214^{ko} background.

(A) *In vivo* tumor growth and metastatic dissemination of EO771 cells injected subcutaneously in miR-214^{wt} and miR-214^{ko} syngeneic mice, 30 days post-inoculation. Relative primary tumor weight (measured in grams) and number of lung metastases is shown in the graphs as mean ± SEM, for the indicated number of mice (n). Representative images of lung metastases are also shown. **(B)** Analysis of S100b expression in a lung lobe derived from B16-F10-xenografts grown in miR-214^{wt} and miR-

214^{ko} mice by qRT-PCR analysis. Results were calculated as fold changes (mean ± SD of triplicates) relative to the median of samples for the indicated number (n) of mice, normalized on 18S rRNA levels. SD=standard deviation. *P≤0.05; **P≤0.01; ***P≤0.001.

Figure S12

miR-214-rich extracellular vesicles (EVs) promote melanoma cell dissemination.

(A) Transendothelial migration through a HUVECs monolayer on top of a porous membrane for B16-F10 cells pretreated for 24h with EVs derived from miR-214^{wt}, miR-214^{over} or miR-214^{ko} CAFs. Top: representative pictures of HUVECs monolayer before plating and B16-F10 migrated cells. Bottom: results expressed as mean±SEM of the area (pixels) covered by migrated cells. 3 independent experiments (with quadruplicates) were performed and pooled results are shown. **(B)** miR-214 expression levels in blood-isolated EVs of miR-214^{ko} mice 15 minutes after tail vein injections of EVs derived from miR-214^{wt}, miR-214^{over} and miR-214^{ko} CAFs, measured by qRT-PCR analysis. Results are shown as fold changes (mean±SD of triplicates) relative to the median of all samples, normalized on U6 for the indicated number (n) of mice. EVs= extracellular vesicles; CAFs=cancer associated fibroblasts; SEM= standard error of mean; SD=standard deviation. *P≤0.05; **P≤0.01; ***P≤0.001.

Figure S13

Correlations bewteen miR-214 and IL6/STAT3 axis in human tumors.

(A-B) Correlations between miR-214 expression and different IL-6 and Stat3-related signatures in TCGA-SKCM (A) or TCGA-BRCA (B) datasets. Samples are presented in plots. Non-significant relationships: p-value>0.05. *AZARE_sig* (5); *DAUER_sig* (6); *ALVAREZ_sig* (7); *TH_sig* (8); *Jak/Stat* (9, 10) M11564; *Stat3_sig_down* (8).

References

1. Onuchic V, Hartmaier RJ, Boone DN, Samuels ML, Patel RY, White WM, et al. Epigenomic Deconvolution of Breast Tumors Reveals Metabolic Coupling between Constituent Cell Types. Cell Rep. 2016;17(8):2075-86.
2. Aran D, Sirota M, Butte AJ. Systematic pan-cancer analysis of tumour purity. Nat Commun. 2015;6:8971.
3. Liu J, Lichtenberg T, Hoadley KA, Poisson LM, Lazar AJ, Cherniack AD, et al. An Integrated TCGA Pan-Cancer Clinical Data Resource to Drive High-Quality Survival Outcome Analytics. Cell. 2018;173(2):400-16 e11.
4. Carter SL, Cibulskis K, Helman E, McKenna A, Shen H, Zack T, et al. Absolute quantification of somatic DNA alterations in human cancer. Nat Biotechnol. 2012;30(5):413-21.
5. Azare J, Leslie K, Al-Ahmadie H, Gerald W, Weinreb PH, Violette SM, et al. Constitutively activated Stat3 induces tumorigenesis and enhances cell motility of prostate epithelial cells through integrin beta 6. Mol Cell Biol. 2007;27(12):4444-53.
6. Dauer DJ, Ferraro B, Song L, Yu B, Mora L, Buettner R, et al. Stat3 regulates genes common to both wound healing and cancer. Oncogene. 2005;24(21):3397-408.
7. Alvarez JV, Febbo PG, Ramaswamy S, Loda M, Richardson A, Frank DA. Identification of a genetic signature of activated signal transducer and activator of transcription 3 in human tumors. Cancer Res. 2005;65(12):5054-62.
8. Tell RW, Horvath CM. Bioinformatic analysis reveals a pattern of STAT3-associated gene expression specific to basal-like breast cancers in human tumors. Proc Natl Acad Sci U S A. 2014;111(35):12787-92.
9. Subramanian A, Tamayo P, Mootha VK, Mukherjee S, Ebert BL, Gillette MA, et al. Gene set enrichment analysis: a knowledge-based approach for interpreting genome-wide expression profiles. Proc Natl Acad Sci U S A. 2005;102(43):15545-50.
10. Liberzon A, Birger C, Thorvaldsdottir H, Ghandi M, Mesirov JP, Tamayo P. The Molecular Signatures Database (MSigDB) hallmark gene set collection. Cell Syst. 2015;1(6):417-25.

Fig. S1

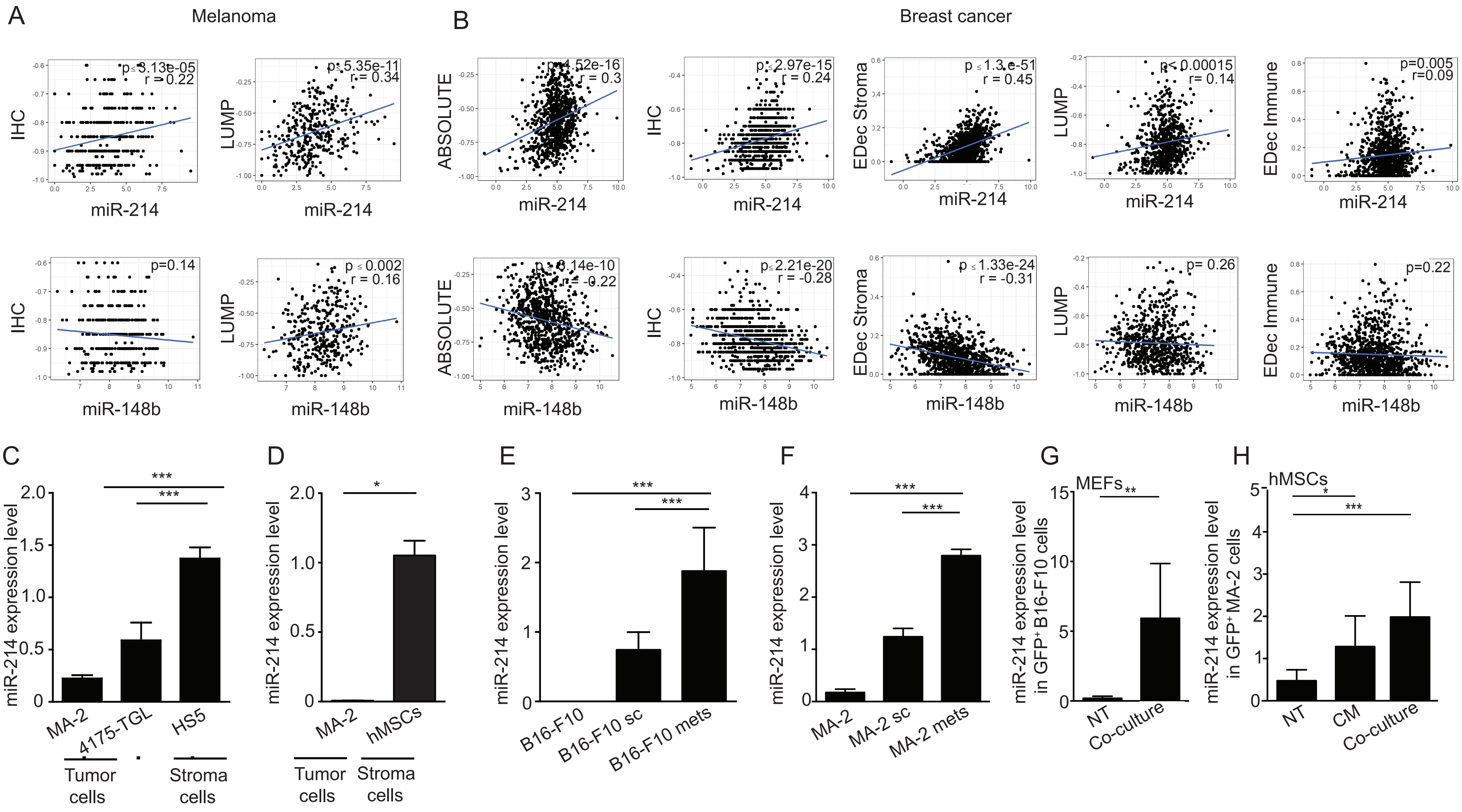
A
Melanoma
B
Breast cancer
IHC
LUMP
ABSOLUTE
EDec Stroma
EDec Immune
miR-214
miR-148b
p≤3.13e-05
r = 0.22
p≤5.35e-11
r = 0.34
p≤4.52e-16
r = 0.3
p≤2.97e-15
r = 0.24
p ≤ 1.3 e-51
r = 0.45
p< 0.00015
r= 0.14
p=0.005
r=0.09
p=0.14
p ≤ 0.002
r = 0.16
p≤8.14e-10
r = -0.22
p≤2.21e-20
r = -0.28
p ≤ 1.33e-24
r = -0.31
p= 0.26
p=0.22
C
D
E
F
G
H
miR-214 expression level
MA-2
4175-TGL
HS5
Tumor cells
Stroma cells
hMSCs
B16-F10
B16-F10 sc
B16-F10 mets
MA-2 sc
MA-2 mets
MEFs
miR-214 expression level in GFP+ B16-F10 cells
NT
Co-culture
miR-214 expression level in GFP+ MA-2 cells
CM

Fig.S2

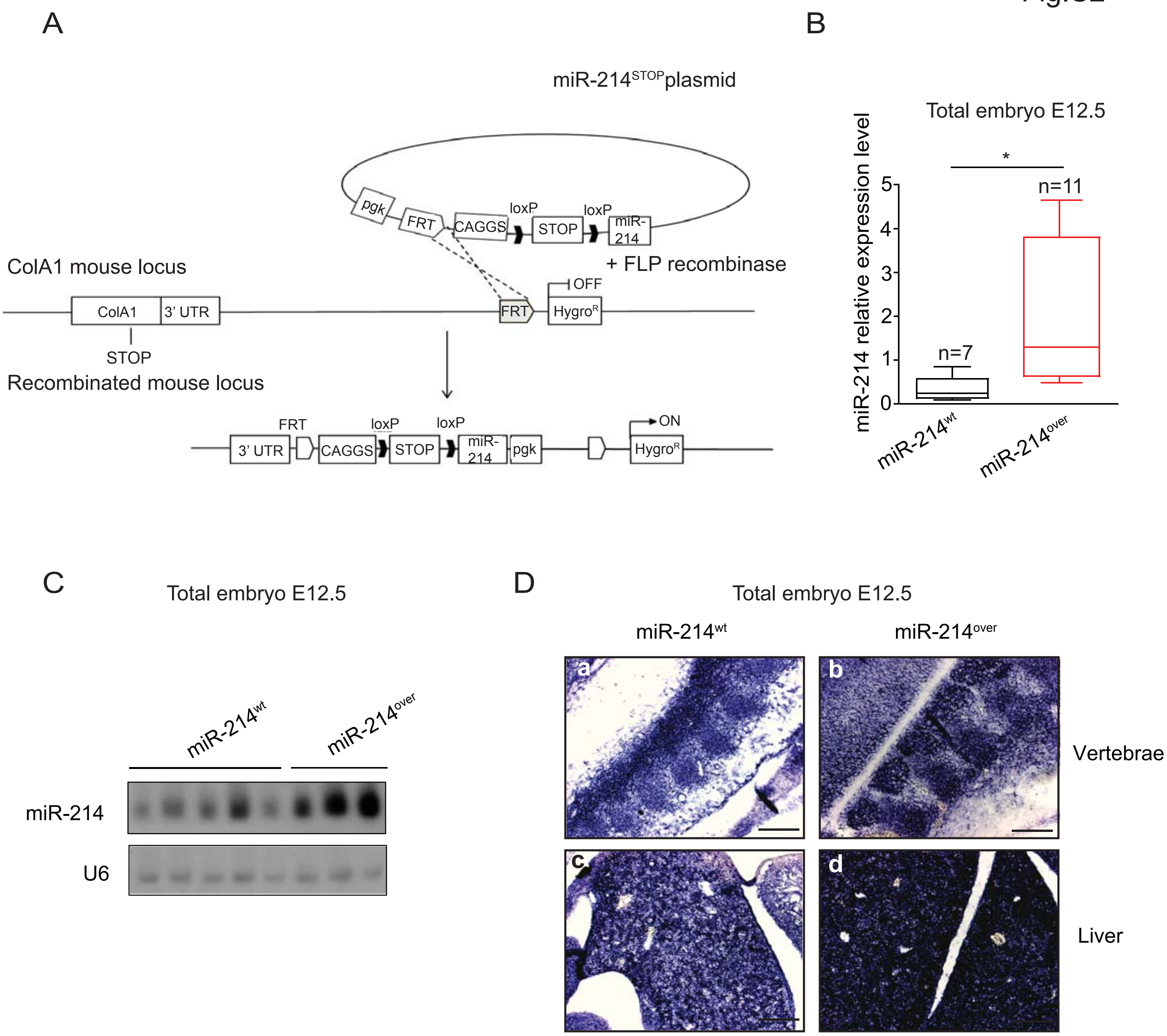

A
miR-214STOPplasmid
pgk
FRT
CAGGS
loxP
STOP
loxP
miR-214
+ FLP recombinase
ColA1 mouse locus
ColA1
3' UTR
STOP
FRT
OFF
HygroR
Recombinated mouse locus
3' UTR
FRT
CAGGS
loxP
STOP
loxP
miR-214
pgk
ON
HygroR
B
Total embryo E12.5
miR-214 relative expression level
*
n=7
n=11
0
1
2
3
4
5
miR-214wt
miR-214over
C
Total embryo E12.5
miR-214wt
miR-214over
miR-214
U6
D
Total embryo E12.5
miR-214wt
miR-214over
a
b
Vertebrae
c
d
Liver

Fig.S3

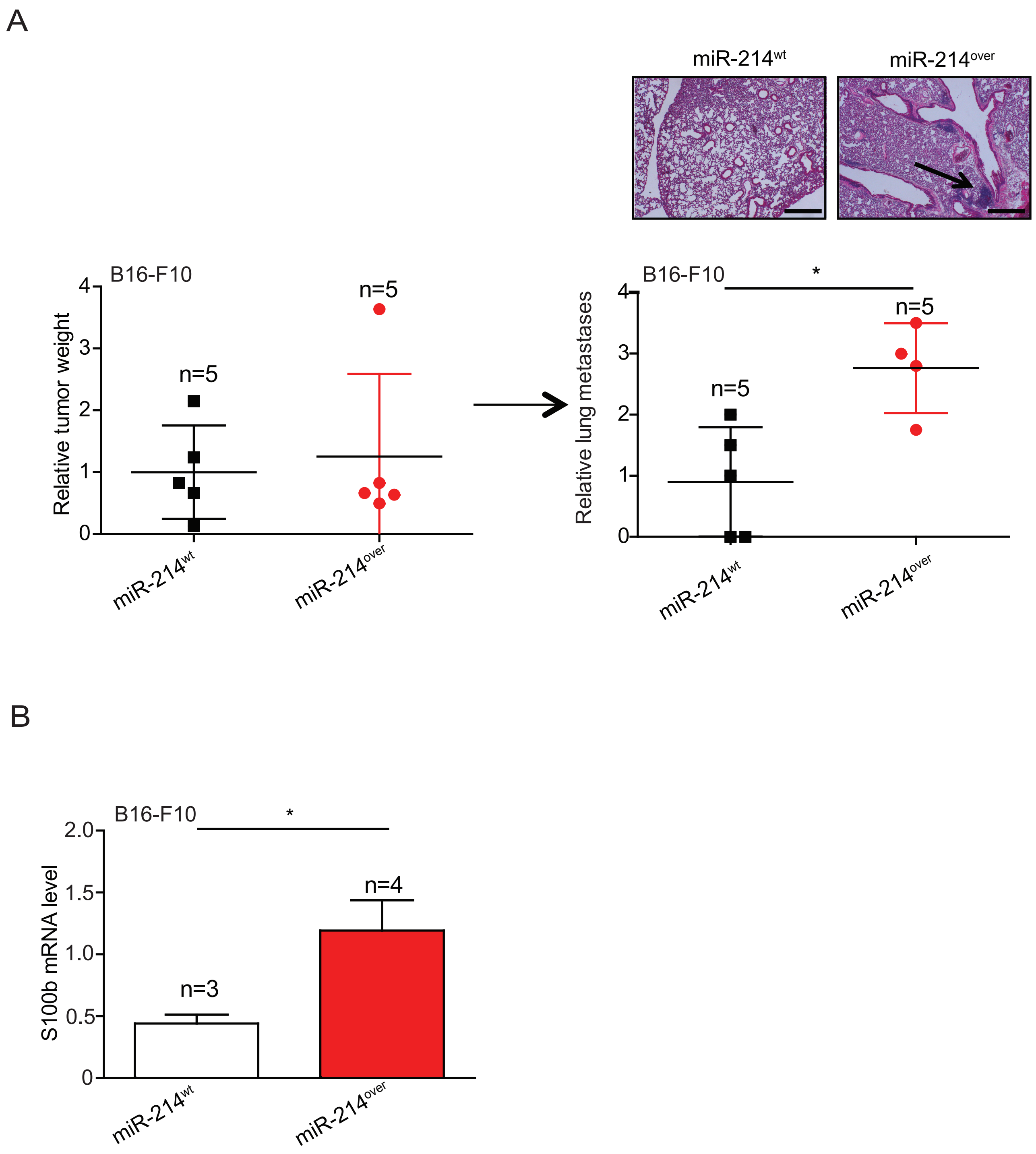

A
miR-214wt
miR-214over
B16-F10
Relative tumor weight
n=5
n=5
miR-214wt
miR-214over
B16-F10
Relative lung metastases
*
n=5
n=5
miR-214wt
miR-214over
B
B16-F10
*
S100b mRNA level
n=4
n=3
miR-214wt
miR-214over

Fig. S4

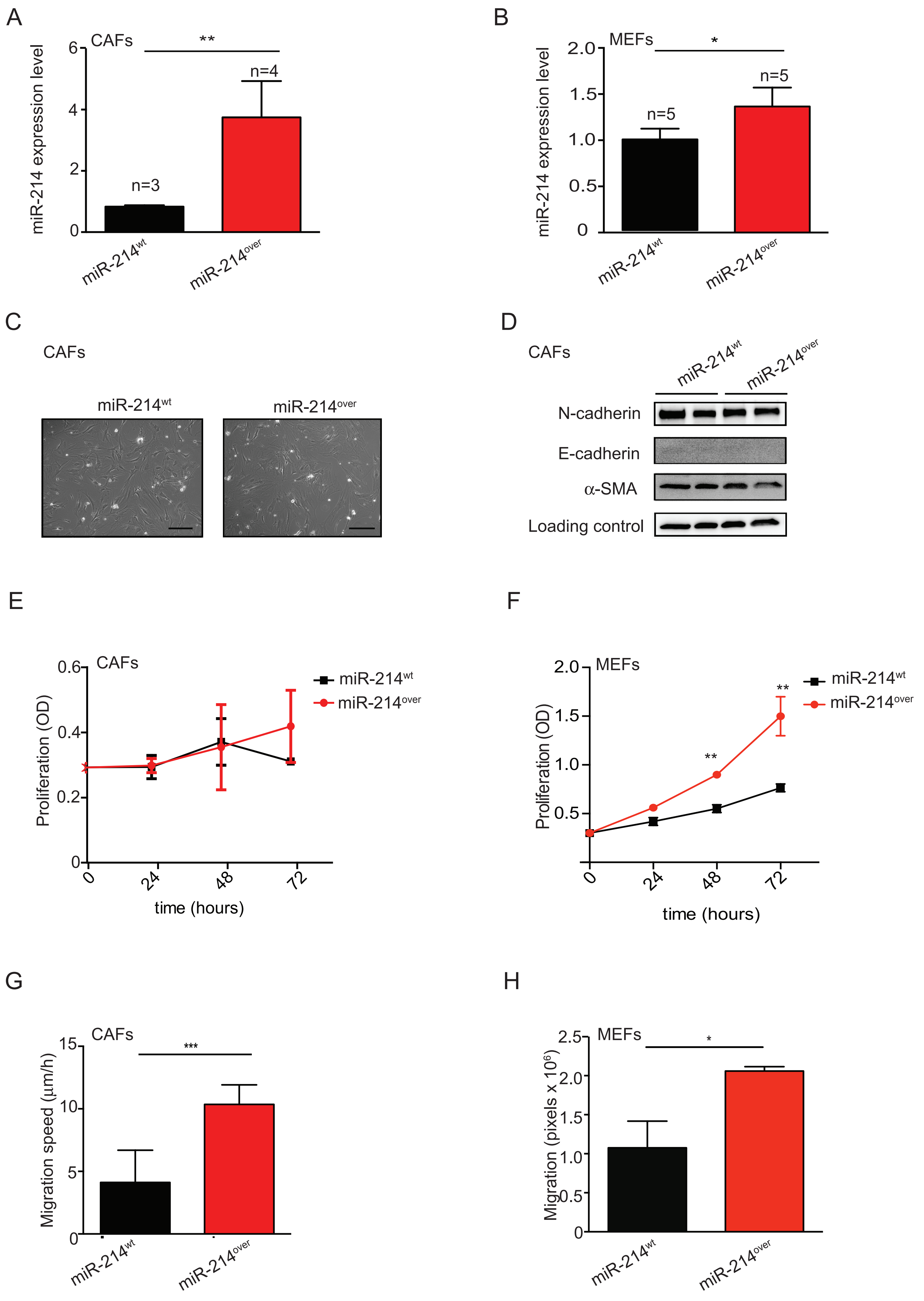
A
CAFs
miR-214 expression level
6
4
2
0
**
n=3
n=4
miR-214wt
miR-214over
B
MEFs
miR-214 expression level
2.0
1.5
1.0
0.5
0
*
n=5
n=5
miR-214wt
miR-214over
C
CAFs
miR-214wt
miR-214over
D
CAFs
miR-214wt
miR-214over
N-cadherin
E-cadherin
α-SMA
Loading control
E
CAFs
Proliferation (OD)
0.6
0.4
0.2
0
0
24
48
72
time (hours)
miR-214wt
miR-214over
F
MEFs
Proliferation (OD)
2.0
1.5
1.0
0.5
0
24
48
72
time (hours)
**
**
miR-214wt
miR-214over
G
CAFs
Migration speed (μm/h)
15
10
5
0
***
miR-214wt
miR-214over
H
MEFs
Migration (pixels x 10⁶)
2.5
2.0
1.5
1.0
0.5
0
*
miR-214wt
miR-214over

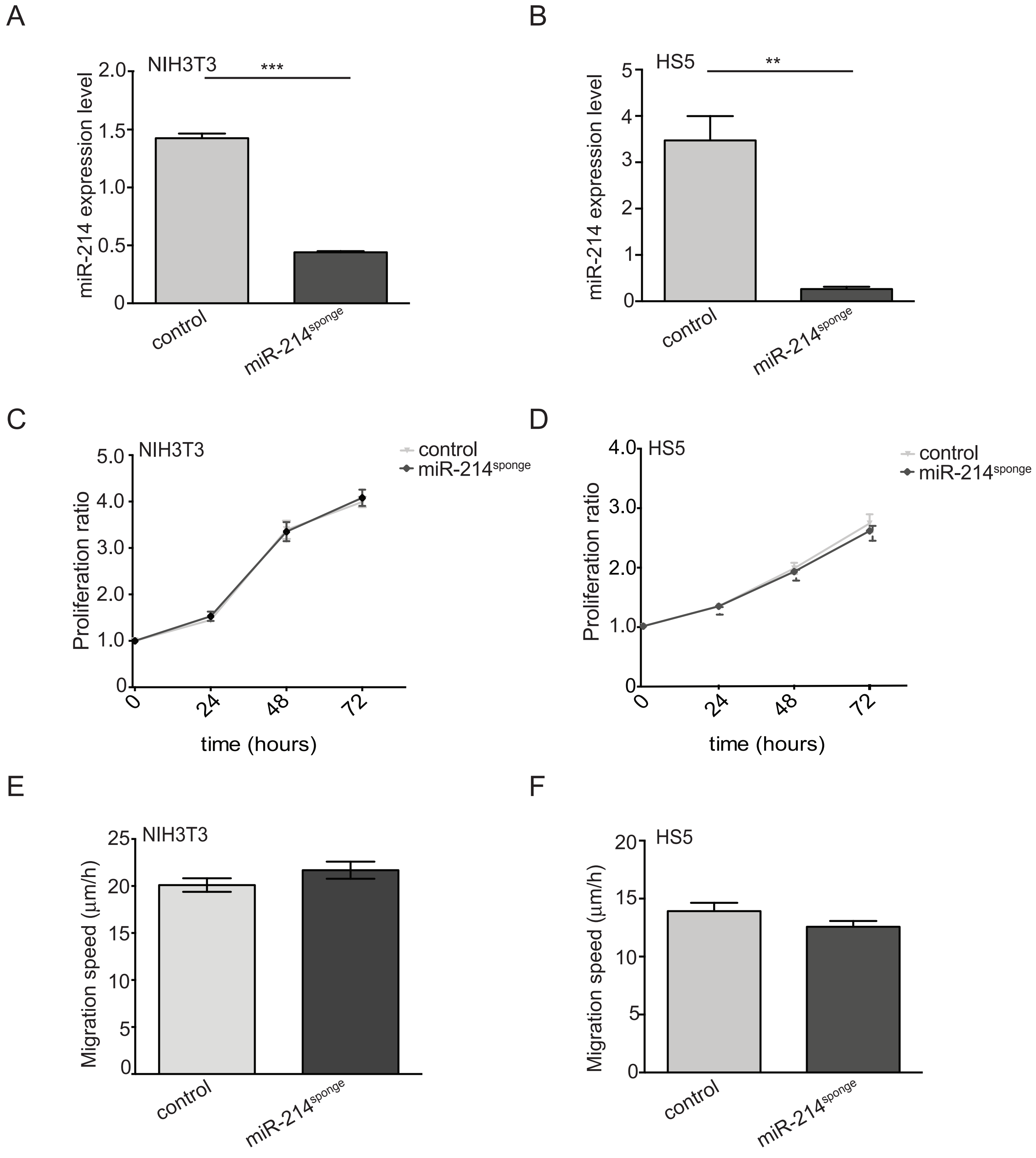
A
NIH3T3
***
miR-214 expression level
2.0
1.5
1.0
0.5
0
control
miR-214sponge
B
HS5
**
miR-214 expression level
5
4
3
2
1
0
control
miR-214sponge
C
NIH3T3
control
miR-214sponge
Proliferation ratio
5.0
4.0
3.0
2.0
1.0
0
0
24
48
72
time (hours)
D
HS5
control
miR-214sponge
Proliferation ratio
4.0
3.0
2.0
1.0
0
0
24
48
72
time (hours)
E
NIH3T3
Migration speed (µm/h)
25
20
15
10
5
0
control
miR-214sponge
F
HS5
Migration speed (µm/h)
20
15
10
5
0
control
miR-214sponge

Fig. S6

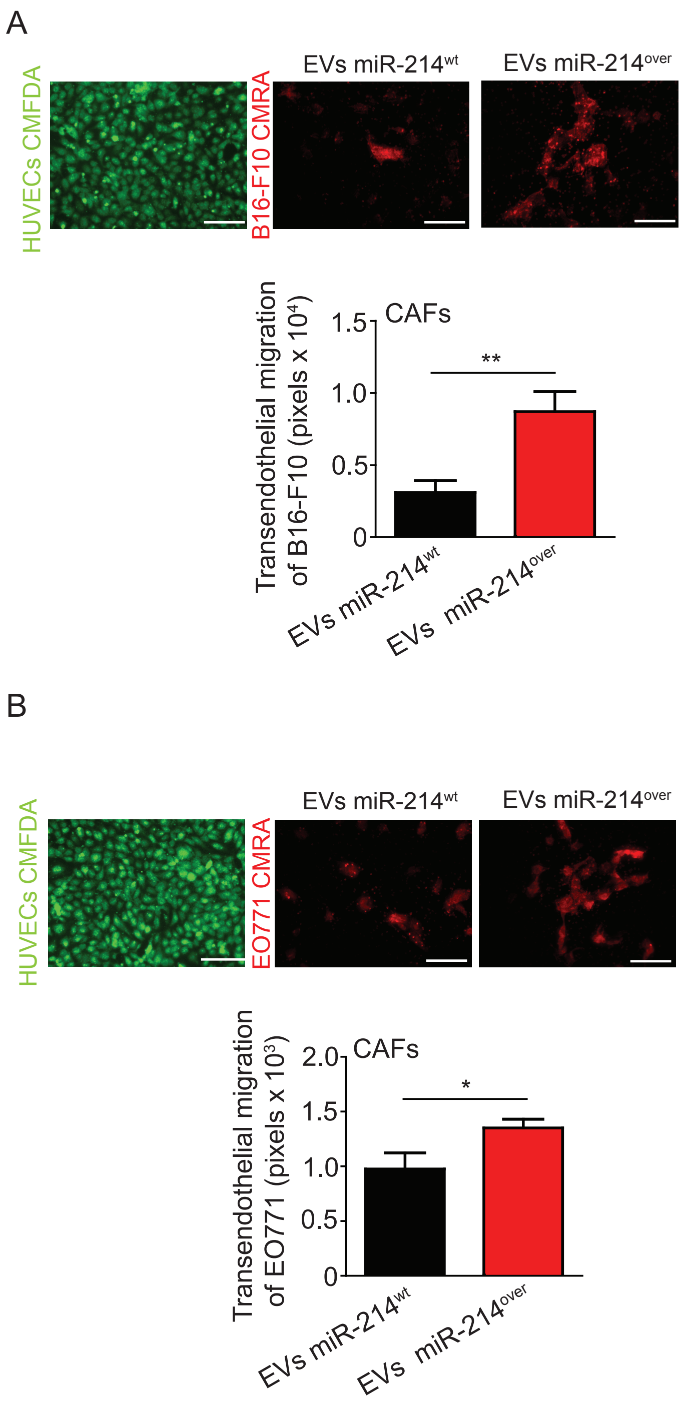
A
HUVECs CMFDA
B16-F10 CMRA
EVs miR-214wt
EVs miR-214over
CAFs
Transendothelial migration of B16-F10 (pixels x 10⁴)
1.5
1.0
0.5
0
**
EVs miR-214wt
EVs miR-214over
B
HUVECs CMFDA
EO771 CMRA
EVs miR-214wt
EVs miR-214over
CAFs
Transendothelial migration of EO771 (pixels x 10³)
2.0
1.5
1.0
0.5
0
*
EVs miR-214wt
EVs miR-214over

Fig. S7

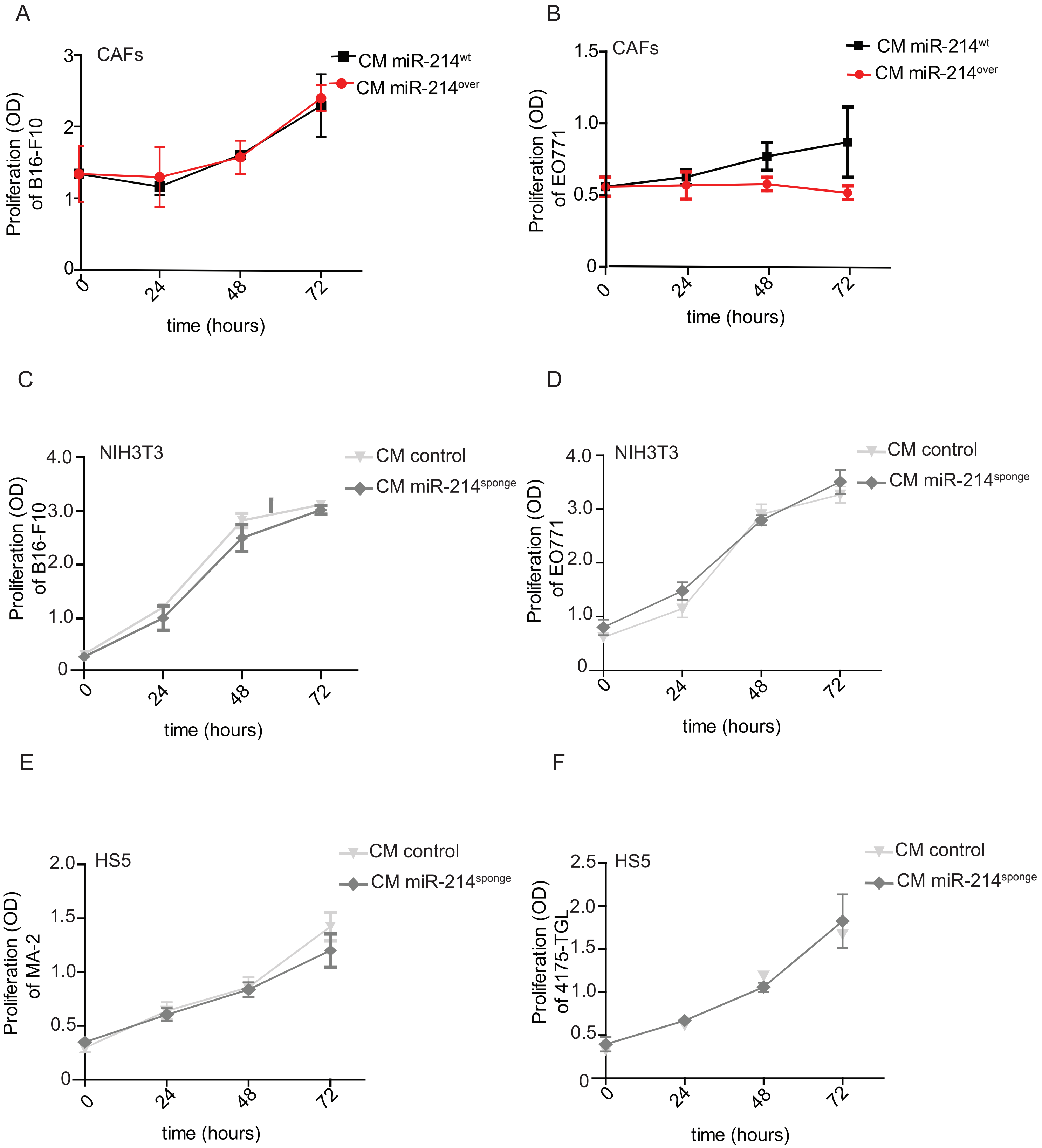
A
CAFs
CM miR-214wt
CM miR-214over
Proliferation (OD) of B16-F10
time (hours)
B
CAFs
CM miR-214wt
CM miR-214over
Proliferation (OD) of EO771
time (hours)
C
NIH3T3
CM control
CM miR-214sponge
Proliferation (OD) of B16-F10
time (hours)
D
NIH3T3
CM control
CM miR-214sponge
Proliferation (OD) of EO771
time (hours)
E
HS5
CM control
CM miR-214sponge
Proliferation (OD) of MA-2
time (hours)
F
HS5
CM control
CM miR-214sponge
Proliferation (OD) of 4175-TGL
time (hours)

Fig. S8

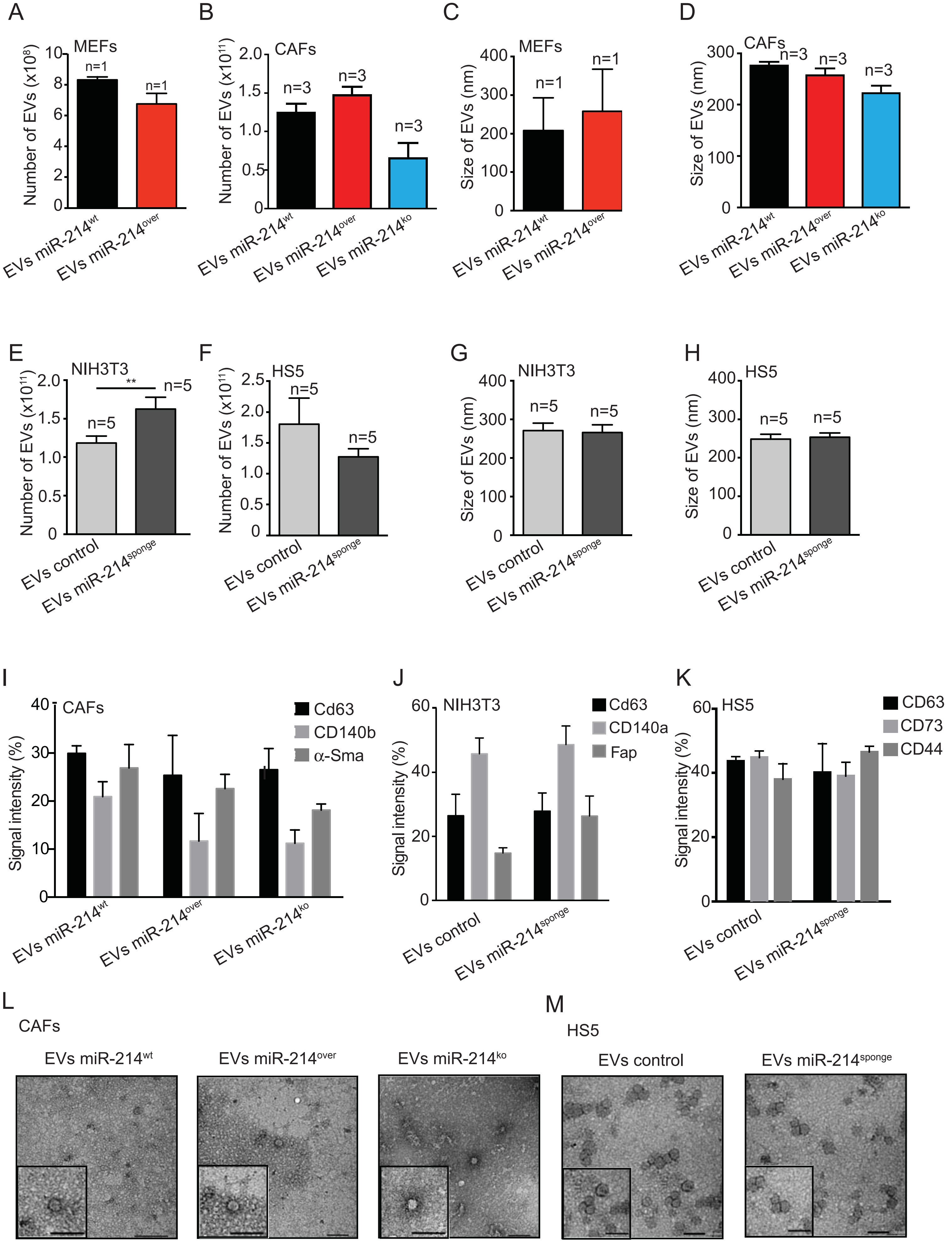
A
MEFs
Number of EVs (x10^8)
EVs miR-214^wt
EVs miR-214^over
B
CAFs
Number of EVs (x10^11)
EVs miR-214^ko
C
Size of EVs (nm)
D
E
NIH3T3
EVs control
EVs miR-214^sponge
F
HS5
G
H
I
Signal intensity (%)
Cd63
CD140b
α-Sma
J
CD140a
Fap
K
CD63
CD73
CD44
L
M

Fig. S9

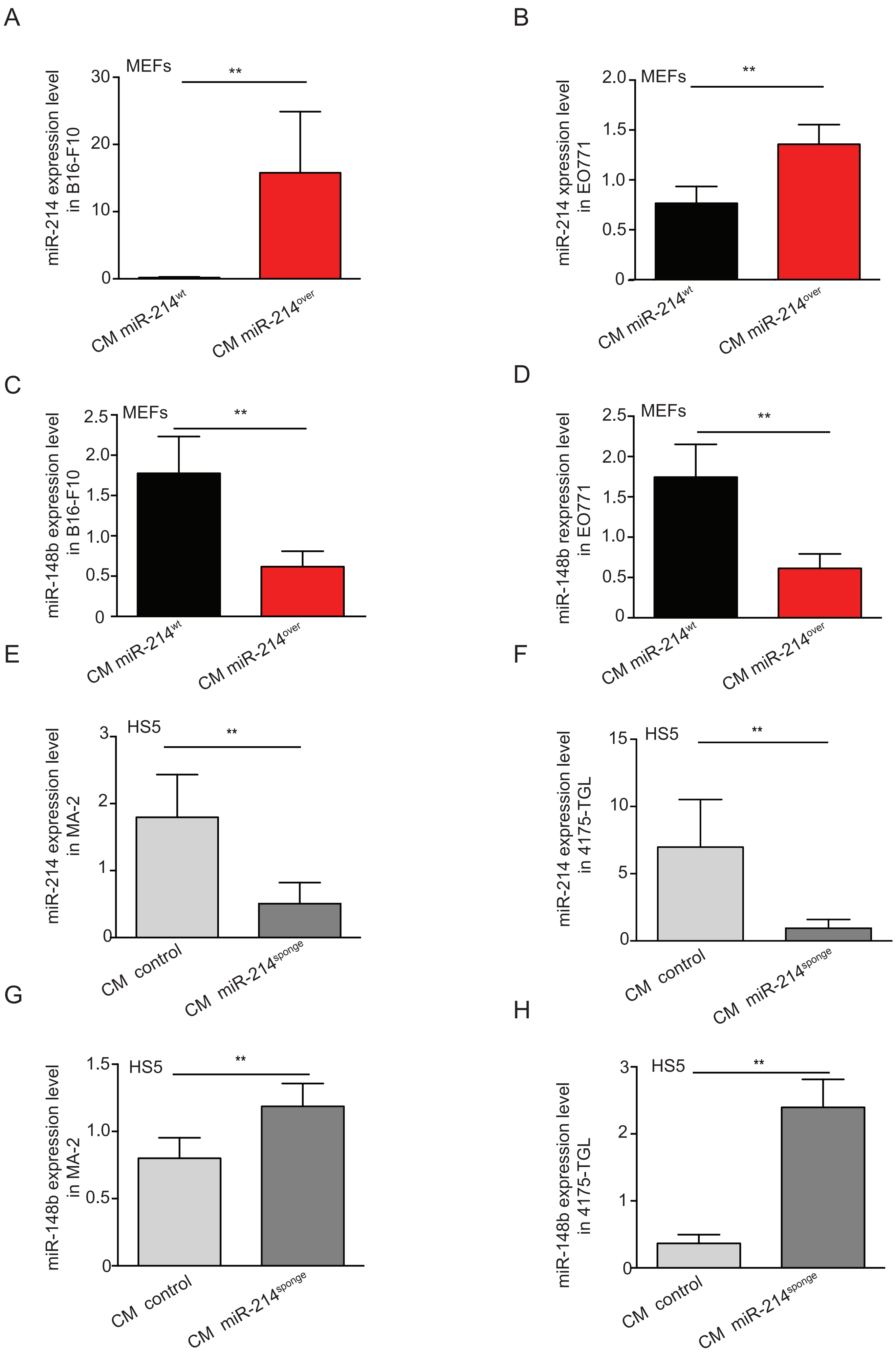
A
MEFs
**
miR-214 expression level in B16-F10
0
10
20
30
CM miR-214wt
CM miR-214over
B
MEFs
**
miR-214 xpression level in EO771
0
0.5
1.0
1.5
2.0
CM miR-214wt
CM miR-214over
C
MEFs
**
miR-148b expression level in B16-F10
0
0.5
1.0
1.5
2.0
2.5
CM miR-214wt
CM miR-214over
D
MEFs
**
miR-148b rexpression level in EO771
0
0.5
1.0
1.5
2.0
2.5
CM miR-214wt
CM miR-214over
E
HS5
**
miR-214 expression level in MA-2
0
1
2
3
CM control
CM miR-214sponge
F
HS5
**
miR-214 expression level in 4175-TGL
0
5
10
15
CM control
CM miR-214sponge
G
HS5
**
miR-148b expression level in MA-2
0
0.5
1.0
1.5
CM control
CM miR-214sponge
H
HS5
**
miR-148b expression level in 4175-TGL
0
1
2
3
CM control
CM miR-214sponge

Fig. S10

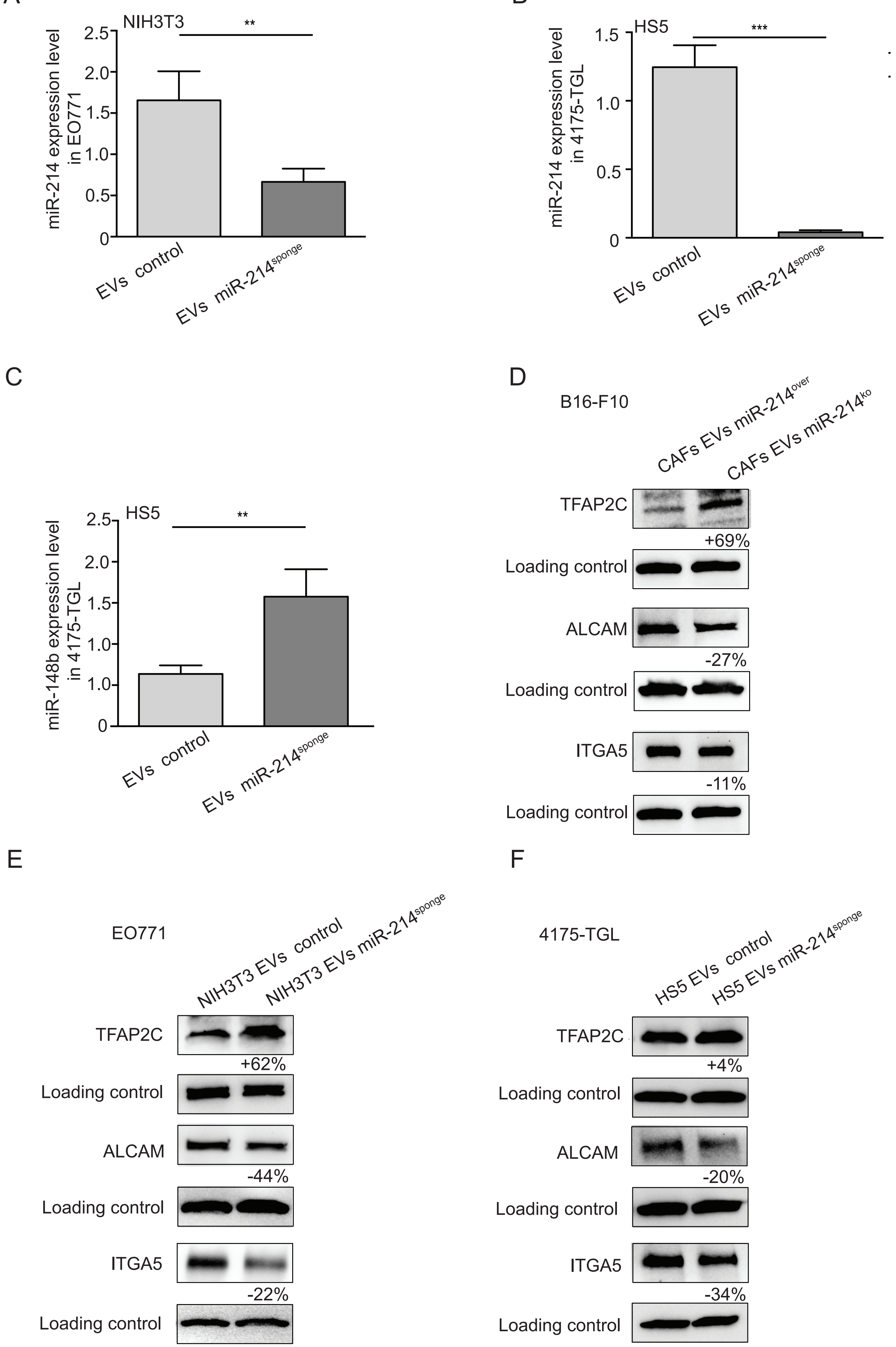

A
NIH3T3
**
miR-214 expression level in EO771
2.5
2.0
1.5
1.0
0.5
0
EVs control
EVs miR-214sponge
B
HS5
***
miR-214 expression level in 4175-TGL
1.5
1.0
0.5
0
EVs control
EVs miR-214sponge
C
HS5
**
miR-148b expression level in 4175-TGL
2.5
2.0
1.5
1.0
1.0
0
EVs control
EVs miR-214sponge
D
B16-F10
CAFs EVs miR-214over
CAFs EVs miR-214ko
TFAP2C
+69%
Loading control
ALCAM
-27%
Loading control
ITGA5
-11%
Loading control
E
EO771
NIH3T3 EVs control
NIH3T3 EVs miR-214sponge
TFAP2C
+62%
Loading control
ALCAM
-44%
Loading control
ITGA5
-22%
Loading control
F
4175-TGL
HS5 EVs control
HS5 EVs miR-214sponge
TFAP2C
+4%
Loading control
ALCAM
-20%
Loading control
ITGA5
-34%
Loading control

Fig. S11

A

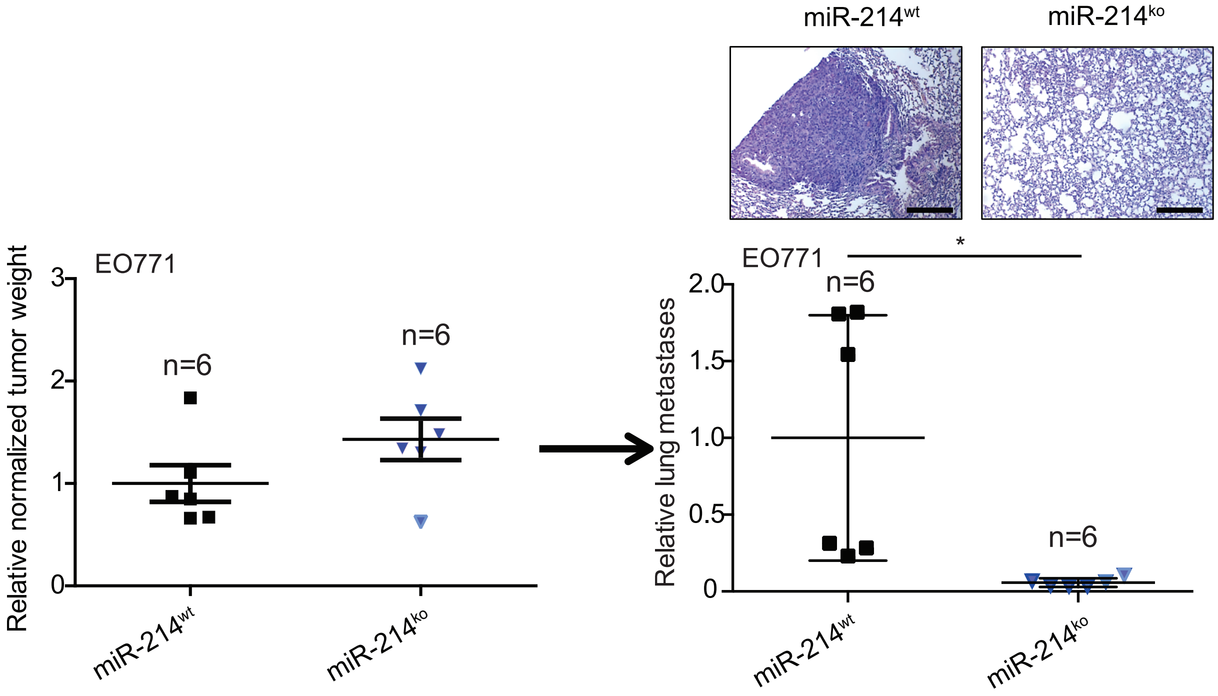
miR-214wt
miR-214ko
EO771
Relative normalized tumor weight
n=6
n=6
miR-214wt
miR-214ko
EO771
*
Relative lung metastases
n=6
n=6
miR-214wt
miR-214ko

B

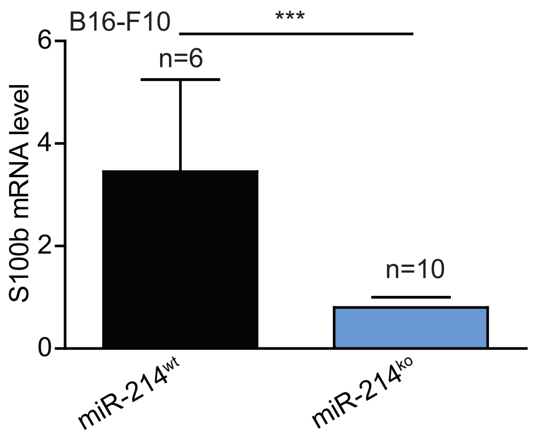
B16-F10
***
S100b mRNA level
n=6
n=10
miR-214wt
miR-214ko

Fig. S12

A

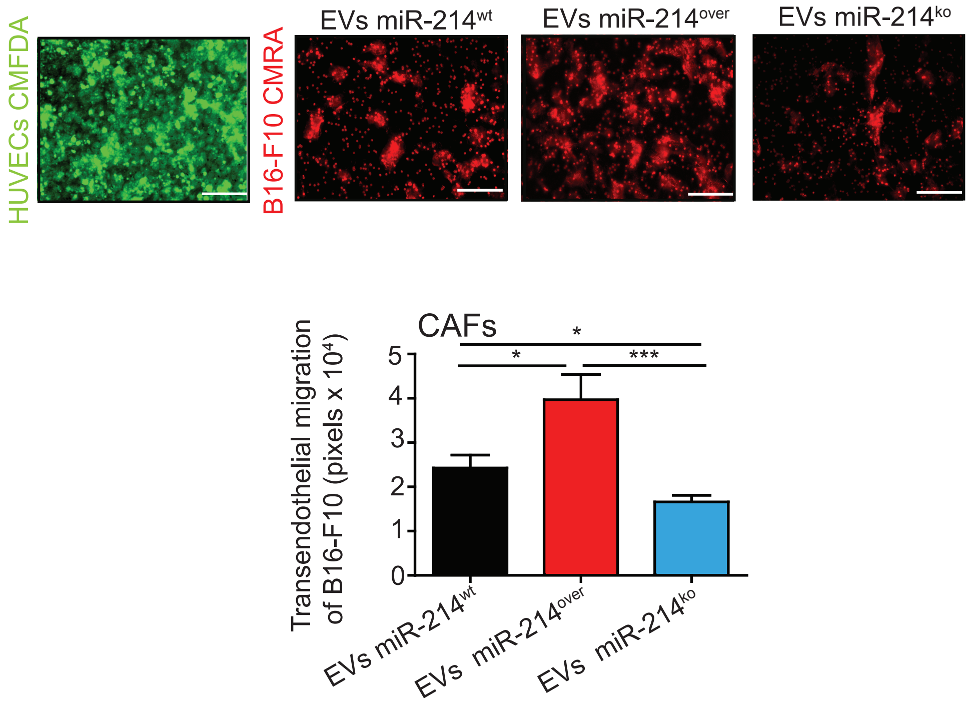

HUVECs CMFDA
B16-F10 CMRA
EVs miR-214wt
EVs miR-214over
EVs miR-214ko
CAFs
Transendothelial migration of B16-F10 (pixels x 10⁴)
0
1
2
3
4
5
*
*
***
EVs miR-214wt
EVs miR-214over
EVs miR-214ko

B

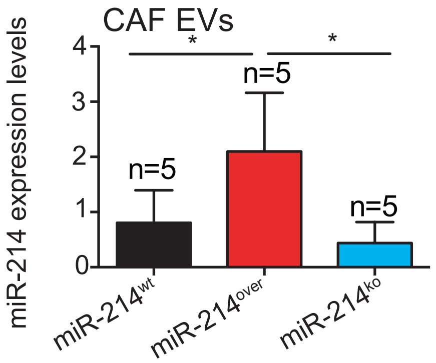

CAF EVs
miR-214 expression levels
0
1
2
3
4
*
*
n=5
n=5
n=5
miR-214wt
miR-214over
miR-214ko

Fig. S13

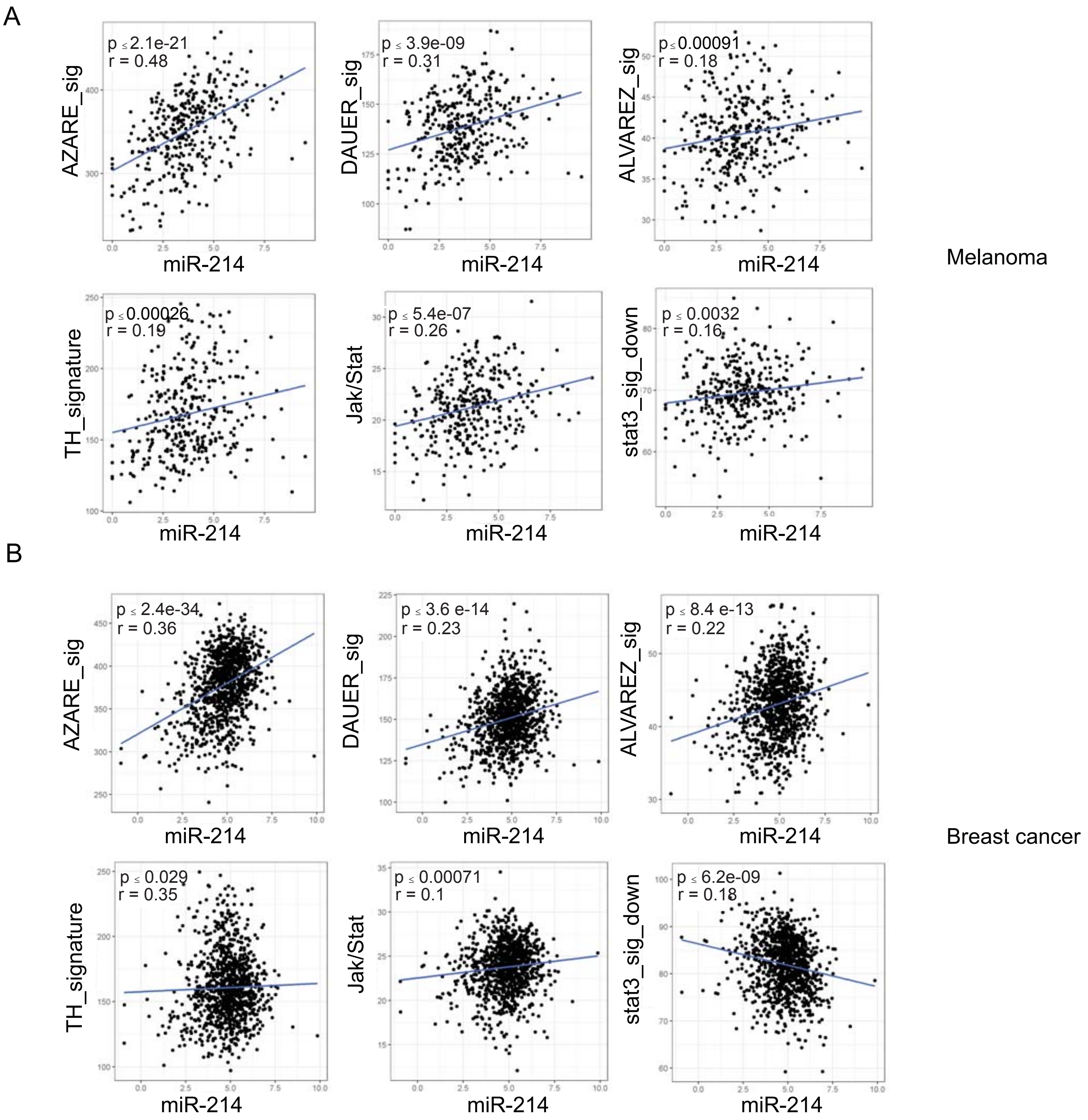

A
p ≤ 2.1e-21
r = 0.48
AZARE_sig
miR-214
p ≤ 3.9e-09
r = 0.31
DAUER_sig
miR-214
p ≤ 0.00091
r = 0.18
ALVAREZ_sig
miR-214
Melanoma
p ≤ 0.00026
r = 0.19
TH_signature
miR-214
p ≤ 5.4e-07
r = 0.26
Jak/Stat
miR-214
p ≤ 0.0032
r = 0.16
stat3_sig_down
miR-214
B
p ≤ 2.4e-34
r = 0.36
AZARE_sig
miR-214
p ≤ 3.6 e-14
r = 0.23
DAUER_sig
miR-214
p ≤ 8.4 e-13
r = 0.22
ALVAREZ_sig
miR-214
Breast cancer
p ≤ 0.029
r = 0.35
TH_signature
miR-214
p ≤ 0.00071
r = 0.1
Jak/Stat
miR-214
p ≤ 6.2e-09
r = 0.18
stat3_sig_down
miR-214
